# Electroacupuncture alleviates myocardial ischemia-reperfusion injury by targeting and inhibiting NLRP3 inflammasome-mediated cardiomyocyte pyroptosis via serum exosomal miR-22-3p

**DOI:** 10.3389/fimmu.2026.1824799

**Published:** 2026-06-01

**Authors:** Jian Xiong, Ying Wei, Yunnan Liu, Fayang Ling, Yi Zhao, Yu Liu, Yuxin Sun, Dehua Li, Mingsheng Sun, Dingjun Cai, Wenchuan Qi, Qianhua Zheng, Fanrong Liang

**Affiliations:** 1 College of Acupuncture, Moxibustion and Tuina, Chengdu University of Traditional Chinese Medicine, Chengdu, Sichuan, China; 2 Sichuan Clinical Research Center for Acupuncture and Moxibustion Medicine, Chengdu, Sichuan, China; 3 Key Laboratory of Acupuncture and Moxibustion for Prevention and Treatment of Geriatric Diseases, Ministry of Education, Chengdu, Sichuan, China; 4 Affiliated Hospital of Chengdu University of Traditional Chinese Medicine, Chengdu, Sichuan, China

**Keywords:** electroacupuncture, exosomes, miR-22-3p, myocardial ischemia reperfusion injury, NLRP3, pyroptosis

## Abstract

**Background:**

Myocardial ischemia-reperfusion injury (MIRI) presents a significant challenge to the effectiveness of reperfusion therapy in severe ischemic heart disease. One of the core pathological mechanisms of MIRI is NLRP3 inflammasome-mediated cardiomyocyte pyroptosis. While electroacupuncture (EA) has demonstrated efficacy in mitigating MIRI, its potential to target and inhibit NLRP3 inflammasome-mediated cardiomyocyte pyroptosis in the context of MIRI remains unclear. Additionally, the mechanism through which local EA may remotely influence the heart to alleviate MIRI is not well understood.

**Objective:**

This study aims to explore the protective effects and molecular mechanisms of EA targeting NLRP3 inflammasome-mediated myocardial cell death in MIRI, focusing on the role of serum exosomes.

**Methods and results:**

An animal model of MIRI was established by ligating the left anterior descending coronary artery, and EA was applied at the Neiguan (PC6) acupoint. Cardiac function, myocardial infarction area, serum myocardial enzymes, inflammatory factors, and myocardial cell pathological changes were assessed using echocardiography, Evans-TTC staining, ELISA, and H&E staining to confirm the therapeutic effect of EA in reducing MIRI. The NLRP3 agonist Nigericin was applied, and the expression levels of key genes and proteins related to NLRP3-mediated apoptosis were measured by Western blotting and RT-qPCR, confirming that EA alleviates MIRI by targeting and inhibiting NLRP3-mediated myocardial apoptosis. The role of serum exosomes in inhibiting NLRP3 inflammasome-mediated cardiomyocyte pyroptosis was then tested in a cardiomyocyte hypoxia/reoxygenation model. Exosomes from serum samples of AMI-PCI patients (with or without standard EA) and from MIRI model rats were isolated. Small RNA sequencing was performed to identify key effector miRNAs post-EA. Our findings revealed that miR-22-3p was significantly upregulated in serum exosomes following EA. Further validation demonstrated that miR-22-3p mitigates MIRI by targeting NLRP3 to inhibit cardiomyocyte pyroptosis, as confirmed through target gene prediction, *in vitro* cell experiments, and *in vivo* animal studies.

**Conclusion:**

This study provides evidence that EA reduces MIRI by upregulating miR-22-3p in serum exosomes, which in turn targets and inhibits NLRP3 inflammasome-mediated cardiomyocyte pyroptosis, thereby reducing severe myocardial injury.

## 1 Introduction

Acute myocardial infarction (AMI) is a life-threatening condition triggered by pathological factors, including atherosclerotic plaques, which lead to sudden and severe reductions, or even complete interruption, of coronary blood flow. This disruption results in sustained ischemia in the affected myocardial regions, ultimately causing myocardial cell necrosis (1). As a major contributor to global disease burden and mortality, AMI poses a significant threat to human health. Percutaneous coronary intervention (PCI) facilitates rapid revascularization of occluded coronary vessels, restoring blood and oxygen supply to the ischemic myocardium. This procedure plays a pivotal role in salvaging viable myocardium, reducing infarct size, and preserving cardiac function, making it the first-line treatment for AMI and other severe ischemic heart conditions according to clinical guidelines (2). However, the restoration of normal perfusion to ischemic myocardium may paradoxically cause secondary damage to myocardial function, metabolism, and structure. This phenomenon, known as myocardial ischemia-reperfusion injury (MIRI), can lead to arrhythmias, heart failure, or sudden death (3, 4). To date, many emerging interventions show great potential in both clinical practice and mechanistic studies. However, effective clinical strategies for preventing or treating MIRI remain lacking, posing a critical barrier to optimizing the benefits of reperfusion therapy in AMI and other severe ischemic heart diseases.

MIRI is a multifaceted process involving energy metabolism disruption, calcium overload, oxidative stress, inflammatory responses, mitochondrial dysfunction and various forms of cell death, with the precise mechanisms yet to be fully understood (5–7). Conventional drugs, exercise, natural products, and ozone therapy target these key pathological processes. These interventions show great therapeutic potential. Studies have shown that ozone-oxygen therapy exerts cardioprotective effects by regulating mitochondrial function and redox balance, and it can also inhibit NLRP3 inflammasome activation and downstream pyroptosis by modulating oxidative stress and inflammatory responses (8, 9). Other studies have also confirmed that regulating calcium overload, inflammation, and cell dysfunction is the core mechanism for treating MIRI (7, 10, 11). The NLRP3 inflammasome serves as a prototypical pattern recognition receptor (PRR) complex within the innate immune system, functioning as a sentinel that detects both pathogen-associated molecular patterns (PAMPs) and damage-associated molecular patterns (DAMPs). Upon activation, it triggers inflammatory cascades that drive tissue injury (12). Pyroptosis, or inflammatory cell death, is a recently identified form of programmed cell death characterized by cell membrane rupture and the release of pro-inflammatory cytokines. It differs from other cell death modalities in terms of pathophysiological morphology and mechanisms (7, 13, 14). Pyroptosis represents a key effector process through which innate immune activation propagates inflammatory tissue damage. The NLRP3 inflammasome-mediated pyroptosis pathway thus establishes a direct mechanistic link between innate immune sensing (via NLRP3) and inflammatory execution (via pyroptosis), positioning this axis as a critical juncture in sterile inflammatory diseases such as MIRI (15–17). Studies have highlighted that NLRP3 inflammasome-mediated pyroptosis is a central pathological mechanism in MIRI (18, 19). NLRP3 activates caspase-1, which cleaves the GSDMD protein, resulting in membrane perforation and the release of IL-1*β* and IL-18, thereby worsening the myocardial inflammatory environment. Inhibition of NLRP3-mediated pyroptosis has been shown to alleviate MIRI in multiple studies (20, 21). Consequently, targeted inhibition of the NLRP3 pyroptosis pathway has emerged as a promising therapeutic approach for MIRI. However, current inhibitors often exhibit off-target effects and toxicity, limiting their clinical application (22). Consequently, identifying safer and more effective strategies to regulate NLRP3 remains a critical area of research.

Acupuncture, a prominent complementary and alternative medical treatment, has demonstrated efficacy in reducing the risk of cardiovascular diseases and providing direct endogenous cardiovascular protection. Among 2,471 systematic reviews on acupuncture indexed in Web of Science from 2000 to 2020, 235 studies (9.5%) focused on acupuncture for cardiovascular conditions, highlighting its substantial clinical attention and solid evidence in this field (23). Our previous clinical studies have shown that acupuncture at points such as Neiguan (PC6) significantly reduces the frequency and severity of angina attacks in patients with chronic stable angina, including those post-PCI (24). Further clinical evidence suggests that acupuncture enhances cardiac function by mitigating inflammatory responses, oxidative stress, and ventricular remodeling, thus safely and effectively reducing MIRI (25). Our basic research has demonstrated that electroacupuncture (EA) at PC6 specifically alleviates MIRI, with mechanisms linked to the regulation of oxidative stress, NOD-like receptors, and the P53 pathway (26). Additionally, EA at PC6 has been shown to improve cardiac function, inhibit inflammatory factor expression and myocardial infarction markers, and alleviate MIRI by regulating alternative splicing and myocardial cell ferroptosis (27, 28). Despite these findings, it remains unclear whether EA exerts anti-inflammatory effects and alleviates MIRI through the targeting and inhibition of NLRP3 inflammasome-mediated myocardial cell pyroptosis.

Exosomes are small vesicles, approximately 50–150 nm in diameter, released into the extracellular matrix upon fusion of intracellular multivesicular bodies with the cell membrane. These vesicles are widely distributed in human body fluids, including blood, milk, saliva, and urine, and can be absorbed by adjacent or distant cells. By carrying bioactive substances, exosomes facilitate intercellular communication and regulation. MicroRNAs (miRNAs), the most abundant nucleic acid bioactive substances in exosomes, play a critical role in regulating post-transcriptional gene expression by targeting mRNAs, offering unique diagnostic and therapeutic potential in cardiovascular diseases (29). Recent studies have extensively explored exosomes and their cargo, such as miRNAs, in the diagnosis, pathogenesis, and treatment of cardiovascular diseases, including MIRI, myocardial infarction, angina pectoris, and heart failure (30–32). In MIRI, influencing circulatory exosomes and their molecular miRNAs plays a pivotal role in exerting myocardial protective effects. Peripheral blood exosomal miR-17-3p, induced by ischemia-reperfusion treatment, alleviates MIRI-associated programmed necrosis by targeting and regulating the expression of TIMP3 (33). Plasma exosomes, in the later phase of remote ischemic preconditioning, enhance PKB and ERK1/2 phosphorylation by transferring miR-126a-3p, activating the RISK signaling pathway, and simultaneously inhibiting the caspase-3-mediated apoptotic signaling pathway, thus mitigating MIRI (34). Plasma exosomal miR-342-5p, induced by long-term exercise, inhibits hypoxia/reoxygenation (H/R)-induced myocardial cell apoptosis by targeting Caspase-9 and Jnk2, providing myocardial protection against MIRI (35). These findings highlight that various stimuli can induce alterations in circulatory exosomal miRNA molecules, contributing to myocardial protection. Acupuncture, as a body surface interventional therapy that does not directly target organs, influences circulatory exosomal miRNAs, which is a key mechanism through which it exerts therapeutic effects on target organs. Previous studies have shown that acupuncture can transmit miR-381 *via* serum exosomes, reducing electrocardiographic ST segment elevation, improving cardiac function, and lowering serum cTn-I levels, thereby alleviating LPS-induced myocardial injury (36). Given that the regulation of circulatory exosomes and their miRNA content plays a critical role in myocardial protection and acupuncture effects, investigating the functional role of serum exosomes following EA is essential. In particular, the mechanism by which EA at local acupoints targets the heart to alleviate MIRI remains unclear.

To address these issues, this study assessed the anti-inflammatory and cardioprotective effects of EA in MIRI rats. The mechanism by which EA alleviates MIRI, through targeting NLRP3 inflammasome-mediated myocardial cell pyroptosis, was further verified. Subsequently, an *in vitro* myocardial cell ischemia/reoxygenation model was employed to confirm that Exo-EA targets NLRP3 inflammasome-mediated myocardial cell pyroptosis. Based on these confirmed effects, exosomal small RNA transcriptome sequencing was performed on Exo-EA from clinical and animal models to identify and experimentally validate key miRNA molecules. It was determined that serum exosomal miR-22-3p, following EA, targets and inhibits NLRP3 inflammasome-mediated myocardial cell pyroptosis, mitigating MIRI-induced inflammatory damage.

## 2 Materials and methods

### 2.1 Animals

Healthy adult male Sprague-Dawley (SD) rats of SPF grade, aged 9–10 weeks and weighing 250–300 g, were obtained from Chengdu Dashuo Experimental Animal Co., Ltd. (license number: SCXK [Chuan] 2020-0030). The rats were housed in the Experimental Animal Research Center of Chengdu University of Traditional Chinese Medicine under controlled conditions(5 per individually ventilated cage, 12-h light-dark cycle, 22-25 °C, 40%-70% humidity, noise < 40 dB)with ad libitum standard feed and sterile drinking water, managed uniformly by professionals. They were provided with standard feed and sterile drinking water, with ad libitum access to both. This study adhered to the “3R” principles for the use of experimental animals, was approved by the Experimental Animal Ethics Committee of Chengdu University of Traditional Chinese Medicine (ethics approval number: 2024072), and conducted in accordance with the guidelines set by the International Guide for the Care and Use of Laboratory Animals and the principles of international laboratory animal care and welfare.

### 2.2 Subjects

The study also complied with Chinese clinical trial regulations and the Declaration of Helsinki, receiving approval from the Ethics Committee of the Affiliated Hospital of Chengdu University of Traditional Chinese Medicine (ethics approval number: 2023KL-065). Inclusion criteria for participants post-PCI for AMI (AMI-PCI) were as follows: (1) Meeting the diagnostic criteria outlined in the 2019 Guidelines for the Diagnosis and Treatment of Acute ST-Segment Elevation Myocardial Infarction by the Chinese Society of Cardiology; (2) Undergoing PCI within 12 hours of admission; (3) Aged 18–85 years, regardless of gender; (4) Cardiac function classified as ≤ Grade II; (5) Voluntary participation with informed consent. A total of 20 AMI-PCI patients were enrolled in the study, randomly assigned to either the EA group or the sham EA (SA) group in a 1:1 ratio. A single-blind design was employed for the clinical trial. Detailed information regarding the clinical trial protocol can be found in a previously published report (37).

### 2.3 MIRI model

The rats were fasted for 12 hours and deprived of water for 4 hours prior to surgery. Anesthesia was induced by intraperitoneal injection of 2% pentobarbital sodium (Sigma-Aldrich, St. Louis, USA) at a dose of 50 mg/kg. After anesthesia, the rats were positioned on the operating table in the supine position. Orotracheal intubation was performed, and the rats were connected to a small-animal ventilator (Nanjing Calvin Biotechnology Co., Ltd., China) for mechanical ventilation, with a respiratory rate set at 70–80 breaths/min, an inspiratory-expiratory ratio of 1:1, and a tidal volume of 12 ml. Electrodes were attached to the PowerLab Labchart system (AD Instruments, Australia) to monitor electrocardiographic changes in the standard chest lead II. A 2-cm transverse incision was made between the 3rd and 4th intercostal spaces at the left sternal border. The subcutaneous tissue was bluntly dissected layer by layer to expose the ribs; the intercostal muscles were separated bluntly, and a thoracotomy retractor was inserted to expose the heart. The pericardium was then carefully torn. A “6-0” suture was used to ligate the left anterior descending artery 2 mm below the left atrial appendage and pulmonary conus, with the ligation depth approximately 1–2 mm and width about 2 mm. Both ends of the suture were threaded through a hose; by pulling the suture ends and pushing the hose, blood flow in the left anterior descending coronary artery was occluded (the sham group underwent the same procedure without coronary ligation). The criteria for successful ischemia included: bulging of the left ventricular anterior wall, clear cyanosis or grayish-white discoloration of the ischemic myocardium in the left ventricular anterior wall, ST-segment elevation greater than 0.1 mV on the electrocardiogram, and intermittent arrhythmia with broadening of the R-wave. After 30 minutes of ischemia, the hemostat was loosened, and the hose was removed to restore blood flow, completing reperfusion. Successful reperfusion was identified by a reduction in left ventricular anterior wall bulging, reddening of the ischemic region, and more than a 50% decrease in ST-segment elevation.

### 2.4 Myocardial cell culture and establishment of hypoxia/reoxygenation model

The rat H9c2 cardiomyocyte line was obtained from the Shanghai Cell Bank, Chinese Academy of Sciences. Cells were cultured in DMEM/F-12 medium (Hyclone, USA) supplemented with 10% exosome-free serum (SBI, USA) and maintained in a cell incubator (Thermo Scientific, USA) at 37 °C in a humidified atmosphere of 5% CO_2_. Cells in the logarithmic growth phase were used for experiments. Upon reaching 80%-90% confluence in T25 flasks, the medium was discarded, and the cells were digested with 0.25% trypsin for approximately 2 minutes. Following cell rounding observed under an inverted microscope, trypsin was removed, fresh medium was added to resuspend the cells, and the cell density was adjusted to 1×10^5^/ml. A 2.0 ml cell suspension was seeded into 6-well plates, ensuring uniform distribution, and cultured overnight at 37 °C in a humidified atmosphere of 5% CO_2_. The medium was then discarded, cells were washed twice with PBS, and the medium was replaced with serum-free low-glucose DMEM. Cells were transferred to a hypoxia chamber (Stem Cell, Canada) with a gas mixture of 1% O_2_, 5% CO_2_, and 94% N_2_ at 37 °C for 5 hours of hypoxic treatment. After hypoxia, the serum-free low-glucose DMEM was replaced with high-glucose DMEM containing 10% exosome-free serum. Cells were then cultured for 1 hour in a cell incubator at 37 °C in a humidified atmosphere of 5% CO_2_ and 95% air for reoxygenation.

### 2.5 Myocardial cell intervention

To verify whether serum exosomes after EA alleviate myocardial cell H/R injury by inhibiting NLRP3-mediated pyroptosis, H9c2 cardiomyocytes were seeded into 6-well plates and cultured until cell confluence reached approximately 70%. The cell experiments were divided into two parts: (1) Establishment of the hypoxia model, which included the control group and the hypoxia model group; (2) The H/R model groups were randomly assigned to the following: H/R+Exo-vector group (incubated with approximately 5.8 × 10^12^ exosome empty vector vesicles), H/R+Exo-SO group (incubated with approximately 5.8 × 10^12^ exosomes from the sham operation [SO] group), H/R+Exo-I/R group (incubated with approximately 5.8 × 10^12^ exosomes from the model group), and H/R+Exo-EA group (incubated with approximately 5.8 × 10^12^ exosomes from the EA Neiguan group). After transfection, each group was cultured in a 37 °C, 5% CO_2_ incubator for 24 hours, followed by the establishment of a myocardial cell H/R model to simulate reperfusion/reoxygenation.

To verify whether miR-22-3p alleviates myocardial cell H/R injury by inhibiting NLRP3-mediated pyroptosis, H9c2 cardiomyocytes were seeded into 6-well plates and cultured until cell confluence reached approximately 70%. The cell experiments were divided into two parts: (1) Establishment of the hypoxia model, which included the control group and the hypoxia model group; (2) The H/R model groups were randomly assigned to the following: H/R+miR-NC group (treated with miR-NC), H/R+miR-22-3p mimic group (treated with miR-22-3p mimic), H/R+miR-22-3p inhibitor group (treated with miR-22-3p inhibitor), and H/R+miR-22-3p mimic+Nig group (treated with miR-22-3p mimic and nigericin [Nig] at 10 μmol/ml). A myocardial cell H/R model was then established to simulate reperfusion/reoxygenation. At the end of the hypoxia period, miR-22-3p inhibitor treatment was applied for 15 minutes, after which the medium was replaced, and reoxygenation was performed in a normal incubator for 6 hours. miR-22-3p mimic (RiboBio, China) and miR-22-3p inhibitor (RiboBio, China) were transfected into H9c2 cardiomyocytes at 100 pmol using a liposomal nucleic acid transfection reagent (Yeasen Biotech, China), respectively.

### 2.6 Electroacupuncture intervention protocol

(1) EA Intervention Protocol in Rats: Rats were captured and restrained in a supine position using a self-made restraining device (China Patent No.: ZL202220028455.4). Both EA at the PC6 acupoint and non-acupoint EA were administered once daily for 20 minutes per session over seven consecutive days. Two stainless steel acupuncture needles (0.25 mm × 13 mm; Suzhou Medical Supplies Factory Company Limited, China) were inserted perpendicularly into the bilateral PC6 acupoints and non-acupoint sites to a depth of approximately 3 mm. An auxiliary needle was inserted 3 mm below the meridian course corresponding to the PC6 acupoint and 3 mm adjacent to the non-acupoint site, respectively, to a depth of 1–2 mm. PC6 Localization: Acupoint positioning was determined according to a prior experimental protocol from our research team (38), located on the medial aspect of the forelimb, in the depression between the radius and ulna bones, 3 mm proximal to the wrist crease. For the non-acupoint EA intervention, two stainless steel needles were inserted at the base of the tail to a depth of 3 mm. This site does not correspond to any traditional acupoint and has been conventionally used as a control non-acupoint site in previous experiments (26). The handles of the acupuncture needle and the auxiliary needle were connected to the positive and negative output terminals, respectively, of a HANS-200 Han’s EA device. Stimulation parameters were set to a current intensity of 1 mA and a frequency of 2 Hz using a dense-disperse wave pattern, with the intensity adjusted to induce slight tremors in the limb connected to the EA device. Rats in groups not receiving EA treatment were restrained once daily for 20 minutes each time, for a total of 7 days.

(2) EA Intervention Protocol in Human Subjects: This study was a randomized controlled trial conducted by our research team; participants were randomly selected from our ongoing RCT population, with 10 subjects in each of the EA group and the sham EA (SA) group. Participants were blinded to group allocation (EA or SA). All treatments were performed in separate single rooms to avoid cross-communication, and acupuncturists used a standardized operation script to minimize unblinding cues. At the end of the 5-day intervention, a participant guessing test was conducted to assess blinding success. This study was registered in the Chinese Clinical Trial Registry (Registration ID: [ChiCTR2400081117]). In addition to standard pharmacological therapy, the first postoperative EA session for patients with AMI who underwent PCI was performed within 12 hours after the procedure for both the EA and SA groups. Treatment was administered once daily for 5 consecutive days, with a needle retention time of 30 minutes per session. EA Group: The PC6, Yinxi (HT6), and Zusanli (ST36) acupoints were selected. Disposable acupuncture needles (0.25 × 25 mm; Suzhou Medical Supplies Factory Company Limited, China) were used for conventional needling. Upon elicitation of the deqi sensation, the needles were connected to a Huatuo brand EA apparatus (Suzhou Medical Supplies Factory Company Limited, China). A dense-disperse wave pattern was applied with a current intensity ranging from 0.2 to 1 mA, adjusted according to patient tolerance, and the needles were retained for 30 minutes. SA Group: The same acupoints as the EA group were selected, but blunt-tipped placebo needle devices were used. After routine skin disinfection, adhesive bases were applied to the skin surface. The needle was inserted vertically through the upper sidewall of the adhesive pad. When pressed, the blunt tip compressed into the pad, generating a pricking sensation without penetrating the skin. The needle was then slightly withdrawn and secured onto the base. The specific acupuncture intervention protocols for both the EA and SA groups were described with reference to a previously published clinical research protocol (37). In the SA group, the EA apparatus was connected to the needles, but no electrical current was delivered, and the needles were retained for 30 minutes. All acupoints were located according to the 2021 National Standard of the People’s Republic of China “Nomenclature and Location of Meridian Points” (GB/T 12346-2021).

### 2.7 Serum exosome extraction and identification

Blood was collected from the abdominal aorta of rats and the brachial artery of human subjects, allowed to stand for 60 minutes, and then centrifuged at 2000 rpm for 10 minutes to separate the serum. The supernatant was collected, aliquoted into cryovials, rapidly frozen in liquid nitrogen, and stored at -80 °C in an ultra-low temperature freezer for future use. Exosomes were extracted using the ultracentrifugation method. Serum samples were quickly thawed in a 37 °C water bath. The samples were transferred to new centrifuge tubes and centrifuged at 2000 × g, 4 °C, for 30 minutes. The supernatant was carefully aspirated and transferred to new tubes, followed by centrifugation at 10,000 × g, 4 °C, for 45 minutes to remove larger vesicles. The supernatant was then filtered through a 0.45-μm filter membrane, and the filtrate was collected. The resulting filtrate was transferred to new centrifuge tubes, and ultracentrifugation was performed at 100,000 × g, 4 °C, for 70 minutes. The supernatant was discarded, and the pellet was resuspended in 10 mL of pre-cooled 1 × PBS. The ultracentrifuge rotor was used again for centrifugation at 100,000 × g, 4 °C, for 70 minutes. After discarding the supernatant, the pellet was resuspended in 100 μL of pre-cooled 1 × PBS to obtain the exosomes. In accordance with the updated guidelines of the Minimal Information for Studies of Extracellular Vesicles 2018 (MISEV2018) published by the International Society for Extracellular Vesicles (ISEV) (39) via TEM and NanoFCM (morphology, particle size, surface markers CD9/CD81).

### 2.8 Serum exosomal RNA extraction and quality control

The lysis buffer was thawed and equilibrated for 5 minutes. A volume of 20 μL of exosomes was mixed with 700 μL of lysis buffer, followed by the addition of 140 μL of chloroform. The mixture was vortexed for 15 seconds and incubated at room temperature for 3 minutes. Subsequently, it was centrifuged at 12,000 × g and 4 °C for 15 minutes. The upper aqueous phase was collected and mixed with approximately 525 μL of absolute ethanol. The mixture was transferred to an adsorption column in two steps, with each step followed by centrifugation at 8,000 × g for 15 seconds at room temperature. The flow-through was discarded. The column was washed sequentially with 700 μL of Buffer RWT and 500 μL of Buffer RPE (each wash step was performed once). A final wash was performed with 500 μL of Buffer RPE, followed by centrifugation at 8,000 × g for 2 minutes. After air-drying the adsorption column, it was transferred to a new collection tube. RNA was eluted with 30 μL of RNase-free water and stored at -80 °C. The concentration and purity of the extracted RNA were assessed using a NanoDrop spectrophotometer (Thermo Scientific NanoDrop 2000, Thermo Scientific, Waltham, Massachusetts, USA). RNA integrity was evaluated using RNA-specific agarose gel electrophoresis (RNA 6000 Nano Kit, Agilent Technologies Inc., California, USA).

### 2.9 Construction of serum exosomal miRNA library and sequencing analysis

The miRNA library was constructed from total RNA using the NEB Next Multiplex Small RNA Library Prep Set for Illumina (New England Biolabs Inc.; Ipswich, Massachusetts, USA). The procedure involved ligating 3’ and 5’ adapters using T4 RNA Ligase, followed by reverse transcription into double-stranded cDNA using Superscript II Reverse Transcriptase. The cDNA fragments were then amplified by PCR, and the target products were size-selected and purified using 15% PAGE gel electrophoresis, resulting in the final library.

Library quality was assessed using the Agilent 2100 Bioanalyzer with the Agilent High Sensitivity DNA Kit (Agilent Technologies Inc., California, USA, Cat# 5067-4626). The total library concentration was measured using the PicoGreen fluorescence-based method (Quantifluor-ST Fluorometer, Promega, Madison, Wisconsin, USA, Cat# E6090; Quant-iT PicoGreen dsDNA Assay Kit, Invitrogen, California, USA, Cat# P7589). The effective library concentration was accurately quantified *via* QPCR (StepOnePlus Real-Time PCR System, Thermo Scientific, Waltham, Massachusetts, USA). Multiple DNA libraries were normalized and pooled in equal volumes. The pooled library was subjected to stepwise dilution and quantification prior to sequencing on an Illumina platform using the PE150 mode. Differentially expressed conserved miRNAs were identified based on the miRNA expression profiles using the edgeR (40) and the DESeq (41) packages. The screening criteria were set as an absolute fold change |log_2_(Fold Change)| > 1 and a statistical significance of P-value < 0.05.

### 2.10 Drug intervention protocol

To investigate the role of NLRP3-mediated cardiomyocyte pyroptosis in the cardioprotective effect of EA against MIRI, Nig (Selleck Chemicals LLC, USA), an NLRP3 agonist, was administered *via* intraperitoneal injection at a dose of 4 mg/kg (42). The injection was given once daily for 7 consecutive days, prior to rat immobilization for EA treatment. An equivalent volume of the drug solvent was administered as a control.

To explore the role of miR-22-3p in MIRI and its involvement in the cardioprotective effects of EA, miR-22-3p ago (Genepharma, China), a chemically modified drug designed to mimic endogenous miRNA and regulate target gene functions, was administered *via* tail vein injection at a dose of 32 OD (approximately 1280 μg). The injection was performed once daily for 7 consecutive days, prior to rat immobilization for EA treatment. An equivalent volume of the drug solvent was administered as a control. Simultaneously, miR-22-3p antago (Genepharma, China), a highly effective inhibitor specifically designed to block endogenous miRNA, was administered *via* tail vein injection at a dose of 160 OD (approximately 6400 μg). This injection was also performed once daily for 7 consecutive days, prior to rat immobilization for EA treatment, with an equivalent volume of physiological saline used as a control.

### 2.11 Assessment of cardiac function in rats by echocardiography

Rats were anesthetized using a small animal gas anesthesia machine (RWD Life Science Company Limited, China). Anesthesia was maintained with 1–2% isoflurane (RWD Life Science Company Limited, China) delivered *via* inhalation at a ventilation rate of 1.5 L/min. The left thoracic area of the rats was shaved to expose a 3 cm × 3 cm skin region. The anesthetized rats were positioned in a supine posture on the operating platform, and an appropriate amount of ultrasonic coupling gel was applied to the left thoracic area. Following a double-blind study design, echocardiographic images were obtained using a small animal color ultrasound imaging system (Philips Epiq 7, China). An ultrasound transducer (parameters: sweep speed 150 mm/s, depth 3 cm, frequency 21 MHz) was placed at the left parasternal region to capture both short-axis views at the level of the papillary muscles and long-axis parasternal left ventricular views. The heart rate was maintained between 300 and 400 beats per minute (bpm). M-mode tracings were recorded from these views. The images were saved, and left ventricular ejection fraction (LVEF) and left ventricular fractional shortening (FS) were calculated. Measurements were taken over three consecutive cardiac cycles and averaged.

### 2.12 Determination of myocardial ischemia and infarct size by Evans blue/TTC staining

Rats were anesthetized with 2% sodium pentobarbital (Sigma-Aldrich, St. Louis, USA) and secured in a supine position on an animal surgical board, with the limbs and head fixed. The chest was reopened through the original incision, and the heart was exposed. The left anterior descending (LAD) coronary artery was re-ligated at the previous site using a 7–0 suture. Then, 2 mL of 2% Evans Blue dye (Sigma-Aldrich, USA) was injected into the left ventricle. After the oral mucosa and distal extremities turned blue, the aortic root was quickly transected, and the heart was excised and rinsed with ice-cold PBS (4 °C). Excess tissue was trimmed, and the surface moisture was blotted dry. The heart was frozen at -80 °C for 15 minutes, while the embedding mold was pre-cooled. The heart was then placed in the mold and vertically sectioned into 1–2 mm thick slices from the apex towards the LAD ligation site. The myocardial slices were immersed in 1% 2,3,5-triphenyltetrazolium chloride (TTC; Sigma-Aldrich, USA) in PBS (pH 7.3) and incubated at 37 °C in the dark for 20 minutes. After incubation, the slices were fixed in 4% paraformaldehyde solution (Lanjeke Technology Company Limited, China) for 24 hours. Following fixation, the slices were arranged in order on glass slides, and images were captured using a digital camera. Normal myocardial tissue stained blue, the area at risk (AAR) appeared red, and the infarct area (IA) appeared white. The areas of the left ventricle (LV), the area at risk (AAR, red + white), and the infarct area (IA, white) were quantified using Image-Pro Plus 6.0 software (Media Cybernetics, USA). The myocardial infarct size was expressed as a percentage of the area at risk (IA/AAR), while the area at risk was expressed as a percentage of the left ventricular area (AAR/LV).

### 2.13 Hematoxylin-eosin staining of myocardial tissue and pathological injury scoring

The lower one-third of the rat myocardial tissue was collected and fixed in 4% paraformaldehyde solution (Lanjeke Technology Company Limited, China) for at least 24 hours. After fixation, the tissue was rinsed with distilled water to remove the fixative, followed by dehydration through a graded ethanol series. The tissue was then embedded, sectioned to approximately 4 μm thickness, and baked. The sections were deparaffinized and cleared by immersion in xylene (Xilong Scientific Company Limited, China) twice, rehydrated through a graded ethanol series, and rinsed with distilled water. The sections were stained with Hematoxylin (Leagene, China) for 10–15 minutes and rinsed with tap water. Differentiation was performed in a 0.5–1% hydrochloric acid-ethanol solution for a few seconds, followed by rinsing with tap water. Bluing was done in warm water for 5–10 minutes, and the sections were thoroughly rinsed with tap water. Counterstaining was carried out with Eosin solution (Leagene, China) for 2 minutes. The sections were then dehydrated through a graded ethanol series, cleared in xylene, and mounted with neutral balsam (Sinopharm Chemical Reagent Company Limited, China). The stained sections were examined under a standard light microscope to evaluate pathological changes, including congestion, blood stasis, hemorrhage, edema, degeneration, necrosis, hyperplasia, fibrosis, organization, granulation tissue formation, and inflammation. Pathological scoring was performed according to criteria established in a previous study (43). The specific scoring criteria were as follows: 0 points—no significant visible injury; 1 point—mild injury; 2 points—moderate injury; 3 points—severe injury; 4 points—extremely severe injury. The average pathological injury score for each rat was calculated from three randomly selected fields of view.

### 2.14 Detection of myocardial enzymes LDH, CK, CK-MB, and myoglobin

Approximately 2 mL of blood was collected from the abdominal aorta of rats and allowed to clot at room temperature for 30 minutes. The sample was then centrifuged at 3000 rpm (approximately 1000 × g) at 4 °C for 10 minutes to separate the serum. The supernatant from processed cell cultures was also collected. Both serum and cell culture supernatant samples were stored at -80 °C for subsequent analysis. The levels of lactate dehydrogenase (LDH), creatine kinase (CK), creatine kinase-MB isoenzyme (CK-MB), and myoglobin (Mb) in the serum and cell culture supernatant were measured using enzyme-linked immunosorbent assay (ELISA) kits (Elara Biotechnology Company Limited, China), strictly following the manufacturer’s instructions for procedure and concentration determination.

### 2.15 Detection of inflammatory factors IL-1*β*, IL-6, TNF-*α*, and MCP-1 in myocardial tissue

The rat hearts were excised, and the ischemic myocardial tissue was dissected and stored at -80 °C for subsequent analysis. The tissue was homogenized, and the levels of Interleukin-1*β* (IL-1*β*), Interleukin-6 (IL-6), Tumor Necrosis Factor-*α* (TNF-*α*), and Monocyte Chemoattractant Protein-1 (MCP-1) in the myocardial tissue were measured using enzyme-linked immunosorbent assay (ELISA) kits (Elara Biotechnology Company Limited, China), strictly following the manufacturer’s instructions.

### 2.16 Western blotting

A small piece of myocardial tissue was cut into fragments and lysed with RIPA buffer containing protease inhibitors (Biosharp, China), followed by homogenization with a cryogenic grinder, centrifugation, and supernatant collection. Total protein concentration was determined using the BCA protein quantification assay kit (Biosharp, China). Protein samples were mixed with loading buffer, denatured at 95 °C for 10 minutes, and separated by 10% SDS-PAGE (Biosharp, China) at a constant 30 mA. Proteins were then transferred onto a PVDF membrane (Merck Millipore, Germany) at 200 mA, which was subsequently blocked with 5% non-fat milk at room temperature for 2 hours on a shaking platform. The membrane was incubated overnight at 4 °C with primary antibodies at the following dilution ratios: NLRP3 (1:500, Affinity, USA), ASC (1:500, Affinity, USA), Caspase-1 (1:2000, Proteintech, USA), GSDMD (1:2000, Proteintech, USA), IL-1*β* (1:500, Bioss, China), IL-18 (1:2000, Proteintech, USA), and *β*-Actin (1:5000, Affinity, USA) as the internal reference. After three 10-minute washes with 1% TBST (Servicebio, China), the membrane was incubated with HRP-conjugated secondary antibody (1:10,000, MULTI SCIENCES, China) at room temperature for 1.5 hours. Following additional washes, the membrane was blotted dry and exposed to ECL chemiluminescent substrate mix (Biosharp, China). Protein bands were visualized and captured using the ChemiScope 6100 gel imaging analysis system and ChemiScope Capture software (both from Qinxiang Scientific Instruments Co., Ltd., China), respectively. Band gray values were analyzed with ChemiScope Analysis software, and the relative expression level of target proteins was calculated as the ratio of the gray value of the target protein band to that of *β*-Actin.

### 2.17 RT-qPCR

Total RNA was extracted from myocardial and serum exosome samples using the Animal Total RNA Isolation Kit (Foregene, China). The RNA concentration and A260/A280 ratio were measured with a micro-volume UV spectrophotometer (Kaio Technology Development Co., Ltd., China). The extracted RNA was then reverse transcribed into cDNA using the 5 × All-In-One MasterMix (with AccuRT Genomic DNA Removal Kit) (Abm, Canada) according to the manufacturer’s instructions. Subsequently, cDNA was subjected to fluorescent amplification using the EvaGreen Express 2 × qPCR MasterMix-No Dye Kit (Abm, Canada) following the provided protocol, with GAPDH or U6 as internal controls. The expression levels of the target mRNA were analyzed and compared using the 2^-ΔΔCt^ method. Primers for the experiment were designed and synthesized by Shanghai Sangon Biotech Co., Ltd., with the specific primer sequences provided in **Supplementary Table 1**.

### 2.18 Analysis of exosome uptake by cardiomyocytes

The PKH26 red fluorescent labeling kit (Sigma, USA) was used to analyze the uptake of myocardial cell exosomes. The PKH26 red fluorescent dye was incubated with freshly extracted exosomes for fluorescent labeling, with an exosome concentration of approximately 2 × 10^12^ cells/L. After labeling, the dye was removed by centrifugation at 200,000 × g for 1 hour, and the labeled exosomes were re-collected. H9c2 cells were seeded, and the experiment was conducted when the cells reached 70%-80% confluence. PKH26-labeled exosomes were added and incubated at 37 °C for 12 hours. The cells were then fixed with 4% paraformaldehyde for 15 minutes, stained with DAPI (SouthernBiotech, USA) for nuclear visualization, and mounted with anti-fluorescence quenching medium. The endocytosis of exosomes in H9C2 cells was observed under a fluorescence microscope (Nikon, Japan).

### 2.19 CCK8 detects the proliferation capacity of cardiomyocytes

Log-phase cardiac muscle cells were seeded at 5 × 10^3^ cells/well in a 96-well plate and cultured for 24 hours. After the cells adhered to the plate, the treatment reagents were added and incubated in the dark. Subsequently, 10 μL of CCK8 working solution (BIOSHARP, China) was added and incubated for 2 hours. The absorbance at 450 nm was measured using a microplate reader.

### 2.20 Double luciferase assay

Bioinformatics software TargetScan was used to predict the binding sites between miR-22-3p and the NLRP3 3’UTR and analyze their target relationship. The 3’UTR sequence of the NLRP3 gene containing the miR-22-3p binding site was amplified by PCR. The NLRP3 3’UTR was then cloned into the pmirGLO luciferase promoter vector (wild-type NLRP3). Mutations were introduced into the NLRP3 3’UTR binding site, and the mutant sequence was cloned into the pmirGLO-NLRP3-MUT plasmid. HEK293-T cells (PROCELL, China) were divided into the following groups: miR-22-3p mimic + pmirGLO-NLRP3, miR-22-3p mimic + pmirGLO-NLRP3-MUT, miR-22-3p mimic NC + pmirGLO-NLRP3, and miR-22-3p mimic NC + pmirGLO-NLRP3-MUT. Following the instructions of Lipofectamine 3000 reagent (Thermo Fisher, USA), miR-22-3p mimic or miR-22-3p mimic NC and the luciferase plasmids pmirGLO-NLRP3 or pmirGLO-NLRP3-MUT were co-transfected into the cells for 48 hours. Luciferase activity was measured using the dual luciferase reporter assay kit (Beyotime, China), with sea cucumber luciferase as an internal control, and fluorescence intensity was detected using a fluorescence detector.

### 2.21 Data statistics and analysis

Data were analyzed using SPSS 27.0 and GraphPad Prism 9.5.0 software. Experimental results are presented as mean ± standard deviation (x ± s). Statistical charts were generated with GraphPad Prism 9.5.0. The normality of the data was assessed using the Shapiro-Wilk test, and the homogeneity of variance was tested with the Levene test. For data meeting the criteria for normal distribution and homogeneity of variance, one-way analysis of variance (ANOVA) was performed for multiple group comparisons, with intergroup differences further analyzed using the Tukey HSD test for pairwise comparisons. If the data did not meet the assumptions of normality or homogeneity of variance, the Kruskal-Wallis nonparametric test was applied for multiple group comparisons, followed by Dunn’s test for *post hoc* analysis. Statistical significance was set at *P* < 0.05, with *P* < 0.01 considered highly significant.

## 3 Result

### 3.1 Electroacupuncture reduces inflammation and alleviates MIRI

**Figure 1:**
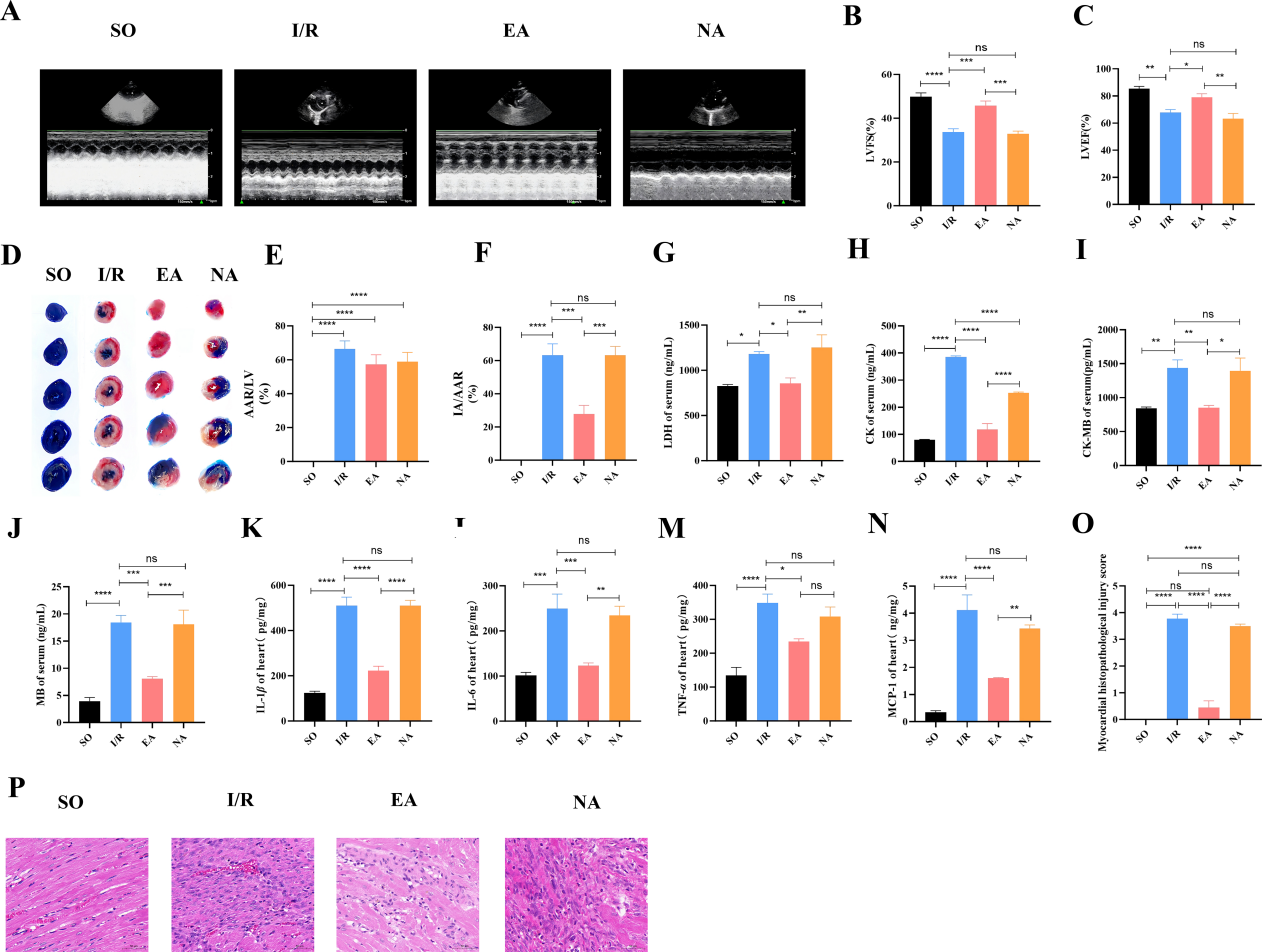
Electroacupuncture reduces inflammation and alleviates MIRI. **(A)** Representative echocardiographic images of the left ventricle. LVFS **(B)** and LVEF **(C)** were measured by echocardiography (n = 6). **(D)** Evans Blue and TTC staining were used to evaluate representative images of the risk and infarction areas following MIRI in each group. The non-ischemic area of the heart section was blue, the infarct area appeared white, and the risk area remained unstained, showing the original myocardial color (red). **(E)** Percentage of risk area, **(F)** infarct area as a percentage of the risk area (n = 5). Serum myocardial enzymes LDH **(G)**, CK **(H)**, CK-MB **(I)**, and MB **(J)** were measured by ELISA. Myocardial tissue inflammatory factors IL-1*β* **(K)**, IL-6 **(L)**, TNF-*α* **(M)**, and MCP-1 **(N)** were measured by ELISA (n = 6). **(O)** Quantitative statistics of myocardial pathological injury score (n = 6). **(P)** Hematoxylin and eosin staining was used to assess pathological damage in myocardial tissue, scale bar: 50 μm. ns *P* > 0.05, ^*^P < 0.05, ^**^P < 0.01, ^***^P < 0.001, ^****^P < 0.0001.

To evaluate the anti-inflammatory protective effects of EA in MIRI, a rat MIRI model was established for *in vivo* experiments. M-mode echocardiography analysis revealed that, compared to the I/R group, EA significantly improved the reduction in LVEF and short-axis shortening fraction (LVFS) induced by MIRI, indicating that EA significantly enhanced cardiac function in MIRI model rats. In contrast, non-acupoint NA did not exhibit similar protective effects on cardiac function (**Figures 1A–C**). Myocardial infarction area was assessed using Evans Blue-TTC staining. Compared to the SO group, the percentage of the myocardial infarction area at risk (AAR/LV) in the I/R, EA, and NA groups was significantly increased to the same extent. EA treatment notably reduced the percentage of myocardial infarction area relative to the area at risk (IA/AAR), whereas NA treatment did not have a similar effect on reducing the infarction area (**Figures 1D–F**). Concurrently, serum levels of lactate dehydrogenase (LDH), creatine kinase (CK), creatine kinase-MB isoenzyme (CK-MB), and myoglobin (Mb) were measured using ELISA to comprehensively assess myocardial injury. EA treatment significantly lowered serum levels of myocardial injury markers (LDH, CK, CK-MB, and Mb), showing a marked advantage over NA treatment. NA treatment only partially reduced CK and CK-MB levels (**Figures 1G–J**). Inflammatory responses play a pivotal role in myocardial survival and repair, being a core component of the MIRI pathological process. Excessive inflammation worsens myocardial injury. In this study, the levels of inflammatory factors in the local myocardial tissue affected by ischemia-reperfusion injury were assessed. The results showed that MIRI caused a substantial increase in the expression of inflammatory factors, such as IL-1*β*, IL-6, TNF-*α*, and MCP-1. These factors act synergistically to promote further inflammatory cell infiltration, creating a vicious cycle that exacerbates myocardial injury. EA treatment effectively suppressed the excessive expression of all inflammatory factors (**Figures 1K–N**), whereas non-acupoint EA treatment did not show a significant reduction in inflammatory factor levels in the ischemic myocardial tissue. These results suggest that the myocardial protective effect of EA may be closely linked to its potent anti-inflammatory properties. Additionally, the pathological morphology of myocardial tissue was examined at the microscopic level using HE staining. In the I/R group, myocardial fibers were disorganized and fractured, with nuclear condensation and dissolution, red cytoplasmic staining, and significant infiltration of inflammatory cells such as neutrophils. There was also marked tissue edema, reflecting typical features of myocardial injury and inflammatory infiltration. In contrast, myocardial tissue treated with EA showed significantly reduced structural damage. The arrangement of myocardial fibers appeared more organized, inflammatory cell infiltration was notably decreased, and tissue edema was alleviated. Quantitative pathological damage scores revealed that the scores for the EA group were significantly lower than those of the I/R group (**Figures 1O, P**). In the non-acupoint EA (NA) group, myocardial tissue damage and inflammatory infiltration did not show similar improvements compared to the EA group. The combined results indicate that, compared to NA, EA treatment exhibits potent anti-inflammatory effects in alleviating MIRI.

### 3.2 Electroacupuncture alleviates MIRI by inhibiting NLRP3 inflammasome-mediated pyroptosis

**Figure 2:**
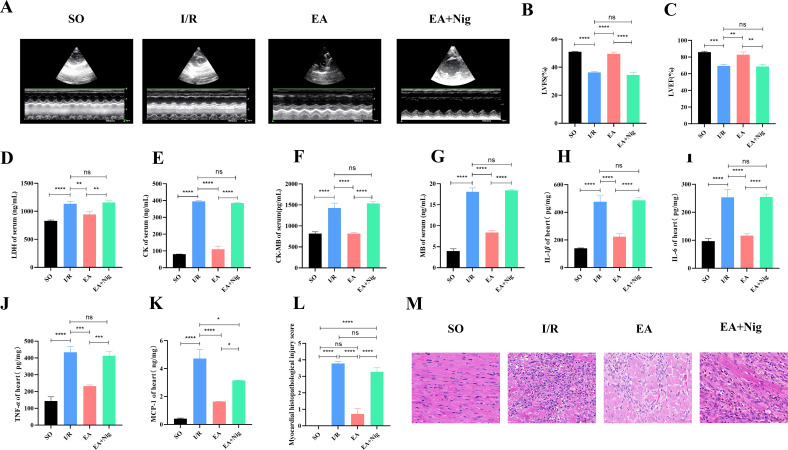
NLRP3 cell apoptosis agonist reverses the effect of electroacupuncture on MIRI injury. **(A)** Representative echocardiographic images of the left ventricle. LVFS **(B)** and LVEF **(C)** were measured by echocardiography (n = 6). Serum cardiac enzymes LDH **(D)**, CK **(E)**, CK-MB **(F)**, and MB **(G)** were analyzed by ELISA (n = 6). Myocardial inflammatory factors IL-1*β* **(H)**, IL-6 **(I)**, TNF-*α* **(J)**, and MCP-1 **(K)** were analyzed by ELISA (n = 6). **(L)** Quantitative statistics of myocardial pathological injury score (n = 6). **(M)** Hematoxylin and eosin staining was used to assess myocardial tissue pathological damage. Scale bar: 50 μm. ns *P* > 0.05, ^*^P < 0.05, ^**^P < 0.01, ^***^P < 0.001, ^****^P < 0.0001.

“Pyroptosis is a pro-inflammatory form of programmed cell death that serves as a key effector mechanism of the innate immune system (44). It is characterized by GSDMD-mediated membrane pore formation, cell swelling, and membrane lysis, leading to the massive release of pro-inflammatory cytokines such as IL-1*β* and IL-18. The NLRP3 inflammasome, a PRR complex of innate immunity, senses DAMPs released during MIRI and triggers pyroptosis (45). Thus, NLRP3-mediated pyroptosis represents a critical juncture where innate immune activation drives inflammatory tissue damage (46). To investigate whether EA exerts anti-inflammatory effects and alleviates MIRI by inhibiting NLRP3-mediated pyroptosis, MIRI model rats were used. Echocardiography revealed that the effects of EA treatment on improving LVFS and LVEF were reversed by the inducer of NLRP3-mediated pyroptosis (**Figures 2A-C**). EA treatment significantly reduced serum levels of myocardial injury markers such as LDH, CK, CK-MB, and Mb. However, this effect was abolished when NLRP3-mediated pyroptosis inducers were co-administered (**Figures 2D-G**). The levels of inflammatory factors, including IL-1*β*, IL-6, TNF-*α*, and MCP-1, in ischemic reperfusion-injured myocardial tissue indicated that EA treatment’s ability to reduce these inflammatory factors was diminished after co-administration of NLRP3-mediated pyroptosis inducers (**Figures 2H-K**). Further pathological analysis revealed that EA treatment significantly reduced myocardial tissue damage scores, alleviated the severity of MIRI, and decreased inflammatory cell infiltration. In contrast, the EA combined with NLRP3-mediated pyroptosis inducer (EA+Nig) group exhibited significantly higher pathological damage scores, with worsened myocardial tissue edema and increased inflammatory infiltration compared to the EA group (**Figures 2L, M**). These results demonstrate that pharmacological activation of NLRP3-mediated pyroptosis abolished the cardioprotective effects of EA treatment. From an innate immunity perspective, this finding indicates that EA exerts its anti-inflammatory and cardioprotective functions largely by restraining the NLRP3 inflammasome-pyroptosis axis—a central innate immune effector pathway. The reversal of EA’s benefits by a pyroptosis inducer underscores that the therapeutic immunomodulation of innate immune-driven pyroptosis is a primary mechanism underlying EA’s protection against MIRI.

**Figure 3:**
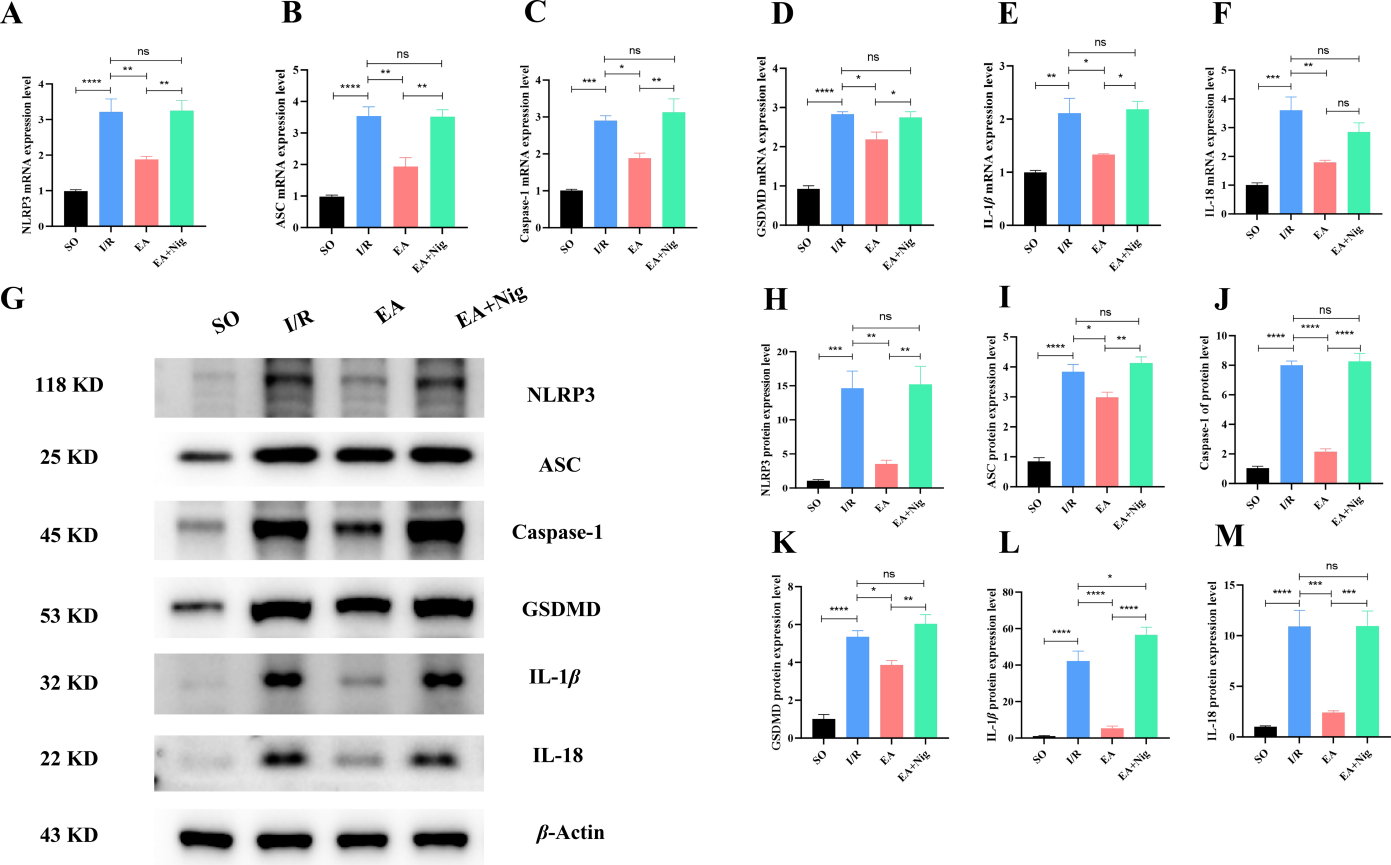
NLRP3 cell pyroptosis agonist reverses the inhibition of electroacupuncture on NLRP3 inflammasome-mediated myocardial pyroptosis mRNA and protein expression. RT-qPCR was used to measure the mRNA expression levels of NLRP3 **(A)**, ASC **(B)**, Caspase-1 **(C)**, GSDMD **(D)**, IL-1*β* **(E)**, and IL-18 **(F)** in myocardial tissue (n = 4). **(G)** Representative image of protein expression levels of NLRP3, ASC, Caspase-1, GSDMD, IL-1*β*, and IL-18 in myocardial tissue by Western blotting. Western blotting was used to measure protein expression levels of NLRP3 **(H)**, ASC **(I)**, Caspase-1 **(J)**, GSDMD **(K)**, IL-1*β* **(L)**, and IL-18 **(M)** in myocardial tissue (n = 5). ns *P* > 0.05, ^*^P < 0.05, ^**^P < 0.01, ^***^P < 0.001, ^****^P < 0.0001.

To elucidate the molecular mechanism by which EA alleviates MIRI through the inhibition of NLRP3-mediated pyroptosis, RT-qPCR and Western blotting (WB) were used to measure the mRNA levels and protein expression of key components in the NLRP3-mediated pyroptosis pathway in myocardial tissue. Compared to the SO group, the mRNA expression levels of core components of the NLRP3 inflammasome (NLRP3, ASC, Caspase-1, GSDMD) were significantly increased in the myocardial tissue from the I/R group. Additionally, the mRNA expression of downstream inflammatory factors, including IL-1*β* and IL-18, was also significantly elevated. In contrast, the mRNA expression of NLRP3, ASC, Caspase-1, GSDMD, IL-1*β*, and IL-18 was significantly reduced in the EA group compared to the I/R group. The NLRP3 agonist reversed the inhibitory effects of EA on the mRNA expression of key components in the NLRP3-mediated pyroptosis pathway (**Figures 3A-F**). WB results further confirmed that, compared to the SO group, the I/R group exhibited significantly higher protein expression levels of NLRP3, ASC, Caspase-1, GSDMD, and the inflammatory factors IL-1*β* and IL-18 in myocardial tissue. In the EA group, however, protein expression levels of these components were significantly reduced compared to the I/R group. The NLRP3 agonist reversed the EA-mediated reduction in protein levels of key components in the NLRP3-mediated pyroptosis pathway (**Figures 3G-M**). These findings reinforce the conclusion that EA alleviates MIRI by inhibiting the NLRP3-mediated pyroptosis pathway, and that the NLRP3 agonist can block this effect.

### 3.3 Serum exosomes after electroacupuncture alleviate hypoxia/reoxygenation injury in cardiomyocytes by inhibiting NLRP3 inflammasome-mediated pyroptosis

Circulating exosomes play a critical role in both the pathology of MIRI and the therapeutic effects of acupuncture. To investigate the impact of serum exosomes on MIRI following EA, serum exosome extraction was performed. Exosomes were purified from serum samples of the SO group, the model (I/R) group, the EA at Neiguan (EA) group, and the NA group using standard sequential density gradient centrifugation and ultracentrifugation methods, 24 hours after the final intervention. Transmission electron microscopy analysis revealed that the particles isolated from serum in all four groups displayed a double membrane structure resembling a cell membrane, with a darker staining on the outer layer and lighter staining on the inner layer. These particles exhibited the typical cup-shaped microvesicle structure, with a round or oval shape (**Supplementary Figure 1A**). Nanoflow cytometry analysis of the size, concentration, and expression levels of specific protein markers in the serum-derived particles showed that the SO group (average particle size 89.57 nm, concentration 3.79E + 10 particles/mL), the model (I/R) group (average particle size 90.13 nm, concentration 6.88E + 10 particles/mL), the EA group (mean particle size 92.09 nm, concentration 3.70E + 10 particles/mL), and the NA group (mean particle size 88.86 nm, concentration 4.35E + 10 particles/mL) showed no significant differences in particle size distribution or serum concentration among the groups (**Supplementary Figures 1B, C**). The particle sizes of serum-derived particles from all four groups ranged from approximately 60 to 150 nm, with the majority concentrated around 70 to 90 nm. Compared to control samples, the relative expression levels of specific protein markers CD9 and CD81 were significantly higher in serum-derived particles from all four groups (**Supplementary Figure 1D**). These results confirm the successful isolation and purification of exosomes from rat serum.

**Figure 4:**
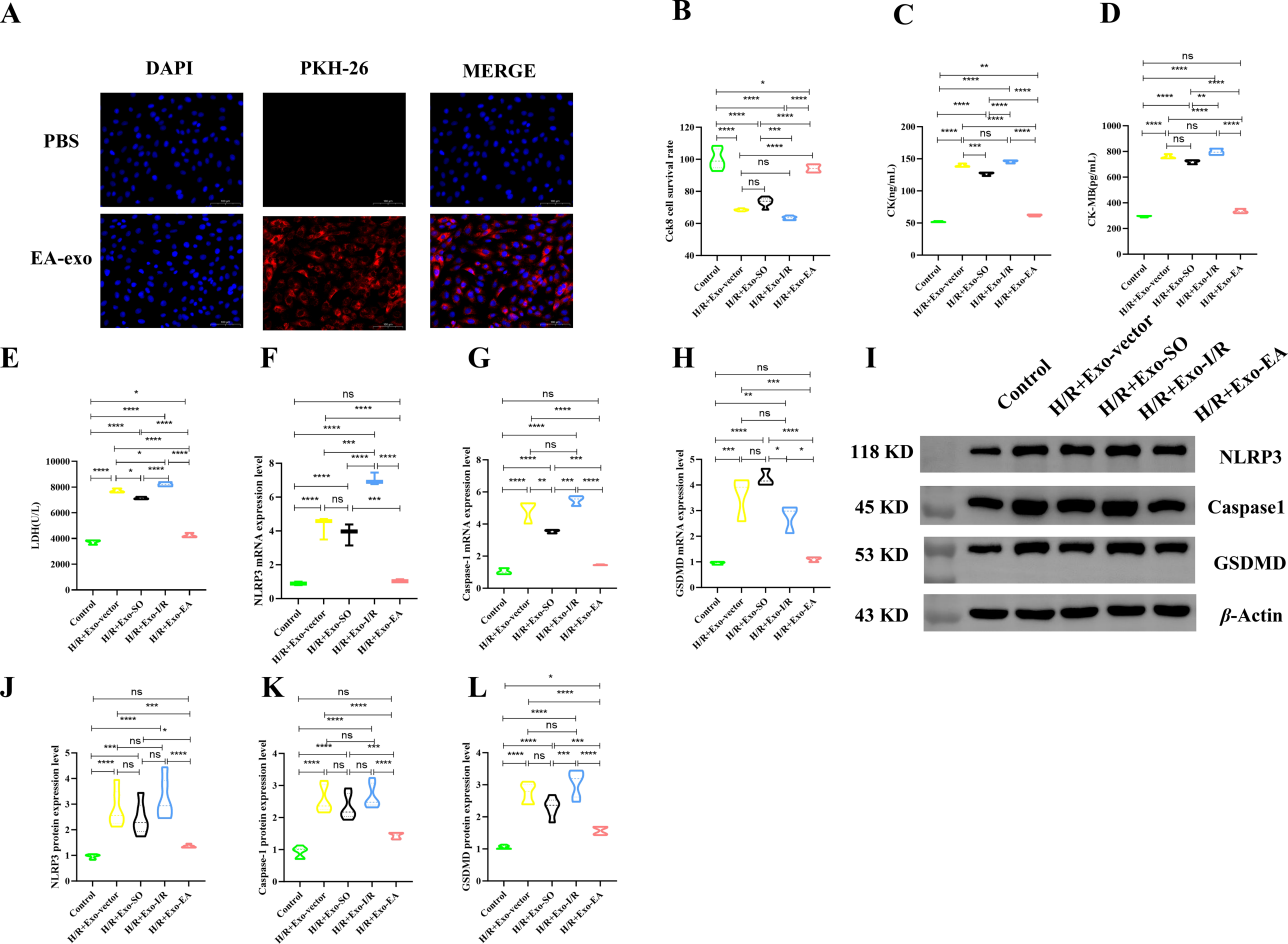
Serum exosomes after electroacupuncture alleviate hypoxia/reoxygenation injury of cardiomyocytes by inhibiting NLRP3 inflammasome-mediated pyroptosis. **(A)** Representative image of serum exosome internalization in cardiomyocytes after PKH-6 labeling post-electroacupuncture. CCK-8 kit for detection of cardiomyocyte survival rate **(B)** n = 6. The cell culture supernatant cardiac enzymes CK **(C)**, CK-MB **(D)**, and LDH **(E)** were analyzed by ELISA (n = 3). RT-qPCR was used to measure the mRNA expression levels of NLRP3 **(F)**, Caspase-1 **(G)**, and GSDMD **(H)** in myocardial tissue (n = 3). **(I)** Representative image of protein expression levels of NLRP3, Caspase-1, and GSDMD in myocardial tissue by immunoblotting. Western blotting was used to detect protein expression levels of NLRP3 **(J)**, Caspase-1 **(K)**, and GSDMD **(L)** in myocardial tissue (n = 6). ns *P* > 0.05, ^*^P < 0.05, ^**^P < 0.01, ^***^P < 0.001, ^****^P < 0.0001.

Next, the role of serum exosomes after EA in alleviating hypoxia/reoxygenation (H/R) injury in cardiomyocytes was evaluated by establishing an *in vitro* cardiomyocyte H/R model. Exosome internalization tracing experiments using PKH-6 fluorescent labeling confirmed that serum exosomes following EA (Exo-EA) were successfully internalized by cardiomyocytes, demonstrating their ability to target and deliver to cardiomyocytes (**Figure 4A**). CCK-8 cell viability assays indicated that treatment with Exo-EA significantly improved the survival rate of myocardial cells subjected to H/R injury, suggesting a protective effect of the exosomes. In contrast, treatment with exosome empty vectors (Exo-vector), serum exosomes from the sham group (Exo-SO), and serum exosomes from the model group (Exo-I/R) did not significantly improve myocardial cell survival after H/R injury (**Figure 4B**). ELISA analysis of the cell culture supernatant revealed that Exo-EA significantly reduced the levels of myocardial injury markers CK, CK-MB, and LDH, alleviating H/R-induced myocardial cell damage (**Figures 4C–E**). However, Exo-vector, Exo-SO, and Exo-I/R treatments did not produce similar effects. RT-qPCR and WB results demonstrated that Exo-EA significantly downregulated the mRNA and protein expression of key pyroptosis molecules NLRP3, Caspase-1, and GSDMD in H/R-injured cardiomyocytes (**Figures 4F–L**). In contrast, treatments with Exo-vector, Exo-SO, and Exo-I/R did not show the same inhibitory effects on NLRP3 inflammasome-mediated pyroptosis-related genes and proteins. These results suggest that Exo-EA significantly alleviate H/R injury in cardiomyocytes by inhibiting the NLRP3 inflammasome-mediated pyroptosis pathway.

### 3.4 Sequencing analysis evidence from experimental animals and clinical subjects indicates that miR-22-3p is the key serum exosomal miRNA by which electroacupuncture alleviates MIRI

**Figure 5:**
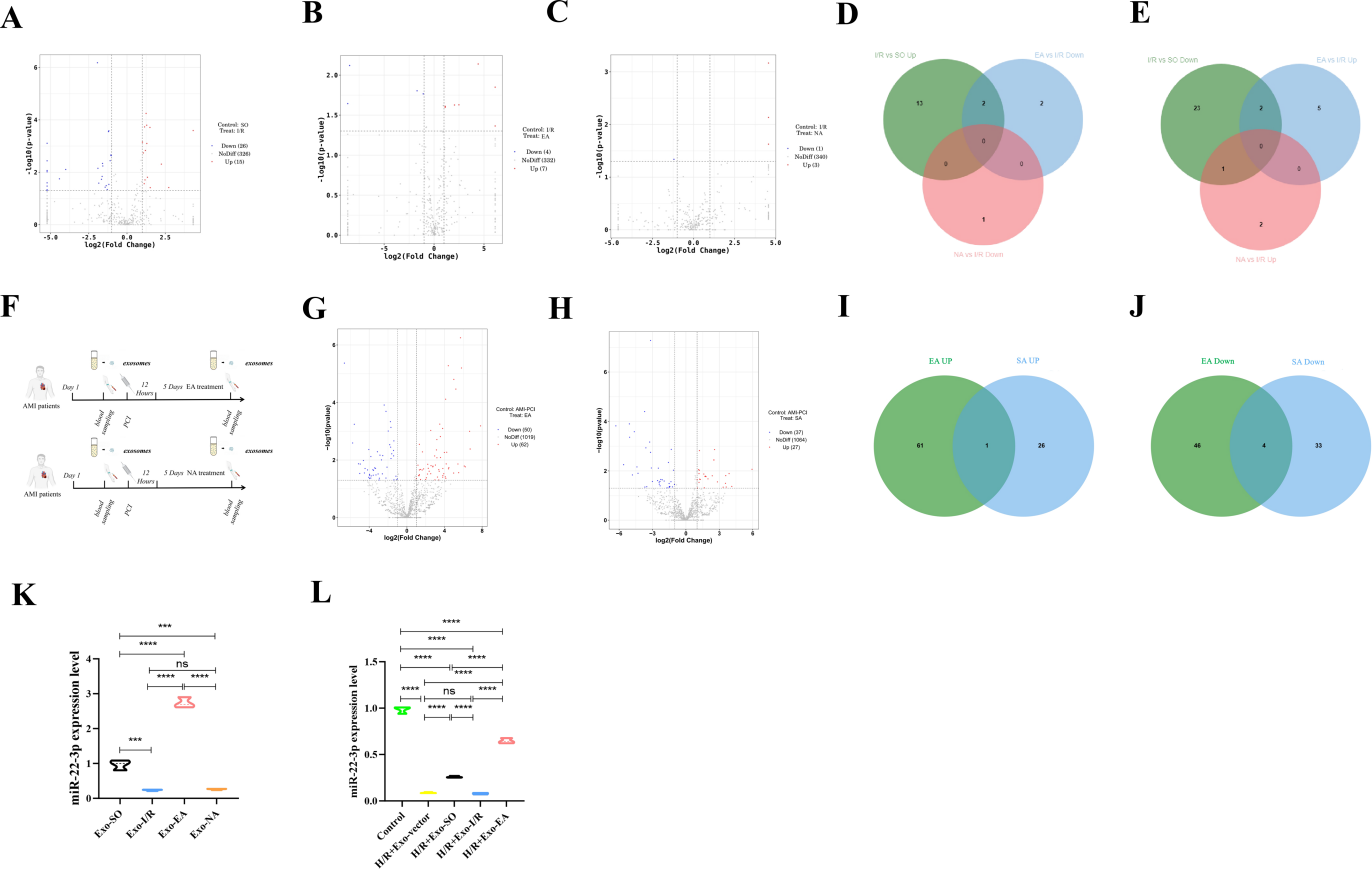
Animal and clinical sequencing evidence suggests miR-22-3p is a key serum exosomal miRNA for electroacupuncture to mitigate MIRI damage. **(A)** Volcano plot of miRNA differential expression in serum exosomes between the I/R and SO groups (n = 3). **(B)** Differential expression of serum exosomal miRNAs between the EA and I/R groups (n = 3). **(C)** Volcano plot of differential miRNA expression in serum exosomes between the NA and I/R groups (n = 3). **(D)** Venn diagram of key serum exosomal miRNAs downregulated by EA. **(E)** Venn diagram of key serum exosomal miRNAs upregulated by EA. **(F)** Schematic of clinical intervention and serum sample collection. **(G)** Volcano plot of miRNA differences before and after clinical trial in the EA group (n = 10). **(H)** Volcano plot of differential miRNA expression in the NA group before and after clinical trial (n = 10). **(I)** Venn diagram of key serum exosomal miRNAs downregulated by EA in clinical trials. **(J)** Venn diagram of key serum exosomal miRNAs downregulated by NA in clinical trials. **(K)** RT-qPCR detection of miR-22-3p expression in serum exosomes (n = 3). **(L)** RT-qPCR detection of miR-22-3p expression after serum exosome incubation (n = 3). ns *P* > 0.05, ^*^P < 0.05, ^**^P < 0.01, ^***^P < 0.001, ^****^P < 0.0001.

To investigate the molecular basis by which Exo-EA alleviates cardiomyocyte H/R injury through the inhibition of NLRP3 inflammasome-mediated pyroptosis, screening and analysis of key miRNAs involved in EA-mediated mitigation of MIRI injury were performed in animal and clinical serum exosomes using small RNA sequencing. By comparing the I/R group with the SO group, differentially expressed miRNAs in serum exosomes associated with myocardial I/R injury were identified, aiming to screen for characteristic biomarkers and disease targets. The Volcano plot of differential miRNA expression in serum exosomes between the I/R and SO groups revealed 41 differentially expressed miRNAs. Among these, 15 miRNAs were upregulated and 26 miRNAs were downregulated in the I/R group compared to the SO group (**Figure 5A**, **Supplementary Table 2**). A comparison between the EA group and the I/R group highlighted miRNAs in serum exosomes that could be regulated by EA in response to MIRI pathology. The EA group showed 4 downregulated and 7 upregulated miRNAs compared to the I/R group (**Figure 5B**, **Supplementary Table 3**). To exclude the non-acupoint stimulation effect of EA, a comparison between the NA group and the I/R group was conducted, revealing miRNAs in serum exosomes regulated by NA in response to MIRI. In the NA group, 4 miRNAs were differentially expressed compared to the I/R group, with 3 miRNAs upregulated and 1 miRNA downregulated (**Figure 5C**, **Supplementary Table 4**). Venn diagram analysis of the miRNAs upregulated in the I/R group compared to the SO group, downregulated in the EA group compared to the I/R group, and downregulated in the NA group compared to the I/R group identified two key downregulated serum exosomal miRNAs in the EA group: miR-1843b-5p and miR-186-5p (**Figure 5D**). Similarly, miRNAs downregulated in the I/R group compared to the SO group, upregulated in the EA group compared to the I/R group, and upregulated in the NA group compared to the I/R group were intersected using a Venn diagram, identifying two key upregulated serum exosomal miRNAs in the EA group: miR-351-3p and miR-22-3p (**Figure 5E**).

To address the species limitations of rat-derived findings, a clinical study was conducted to aid in the screening and validation of experimental conclusions from rat models (**Figure 5F**). AMI-PCI patients were selected as study subjects. In the EA group, serum exosome isolation and small RNA sequencing analysis were performed before PCI treatment and after the completion of EA therapy. An SA treatment group was included as a control, with serum exosome isolation and small RNA sequencing analysis conducted both before PCI treatment and after sham acupoint EA therapy. Transmission electron microscopy analysis revealed that the particles isolated from serum samples in both groups before and after treatment consisted of bilayer membranes resembling cell membranes, with darker staining on the outer layer and lighter staining on the inner layer, showing the typical cup-shaped microvesicle structure in round or oval forms (**Supplementary Figure 2A**). Nanoflow cytometry was used to assess the size, concentration, and specific protein marker expression levels of serum-derived particles. In the EA group (before EA: average particle size 89.5 nm, concentration 1.38E + 11 particles/mL; after EA: average particle size 89.9 nm, concentration 4.25E + 10 particles/mL) and the SA group (before SA: average particle size 91.8 nm, concentration 2.56E + 10 particles/mL; after SA: average particle size 92.0 nm, concentration 1.43E + 10 particles/mL), the relative expression levels of specific protein markers CD9 and CD81 in serum-derived particles were significantly higher before and after treatment compared to control samples (**Supplementary Figure 2D**). These results confirm the successful isolation and purification of exosomes from the serum of subjects in both groups before and after treatment.

By comparing the differential expression of serum exosomal miRNAs before and after EA treatment, a total of 112 differentially expressed miRNAs were identified, with 62 miRNAs upregulated and 50 miRNAs downregulated (**Figure 5G**, **Supplementary Table 5**). Similarly, comparing the differential expression of serum exosomal miRNAs before and after SA treatment revealed 64 differentially expressed miRNAs, with 27 upregulated and 37 downregulated (**Figure 5H**, **Supplementary Table 6**). The upregulated differentially expressed serum exosomal miRNAs before and after EA treatment were intersected with those from before and after SA treatment using a Venn diagram, resulting in 61 key serum exosomal miRNAs upregulated by EA (**Figure 5I**, **Supplementary Table 7**). A comparison of the downregulated differentially expressed serum exosomal miRNAs before and after EA treatment with those from SA treatment, followed by a Venn diagram intersection, identified 48 key serum exosomal miRNAs downregulated by EA (**Figure 5J**, **Supplementary Table 8**).

Comprehensive results from both animal and clinical serum exosome studies identified miR-22-3p as the sole upregulated key serum exosomal miRNA involved in EA’s alleviation of MIRI injury. RT-qPCR analysis of serum exosomes confirmed that miR-22-3p expression was significantly higher in the EA group (**Figure 5K**). Similarly, RT-qPCR analysis demonstrated that cardiac muscle cells treated with purified serum Exo-EA-derived exosomes exhibited significantly elevated miR-22-3p expression (**Figure 5L**). These results from both animal and clinical studies collectively indicate that miR-22-3p is a key serum exosomal miRNA involved in EA’s mitigation of MIRI injury.

### 3.5 miR-22-3p targets and inhibits NLRP3 inflammasome-mediated pyroptosis to alleviate cardiomyocyte hypoxia/reoxygenation injury

**Figure 6:**
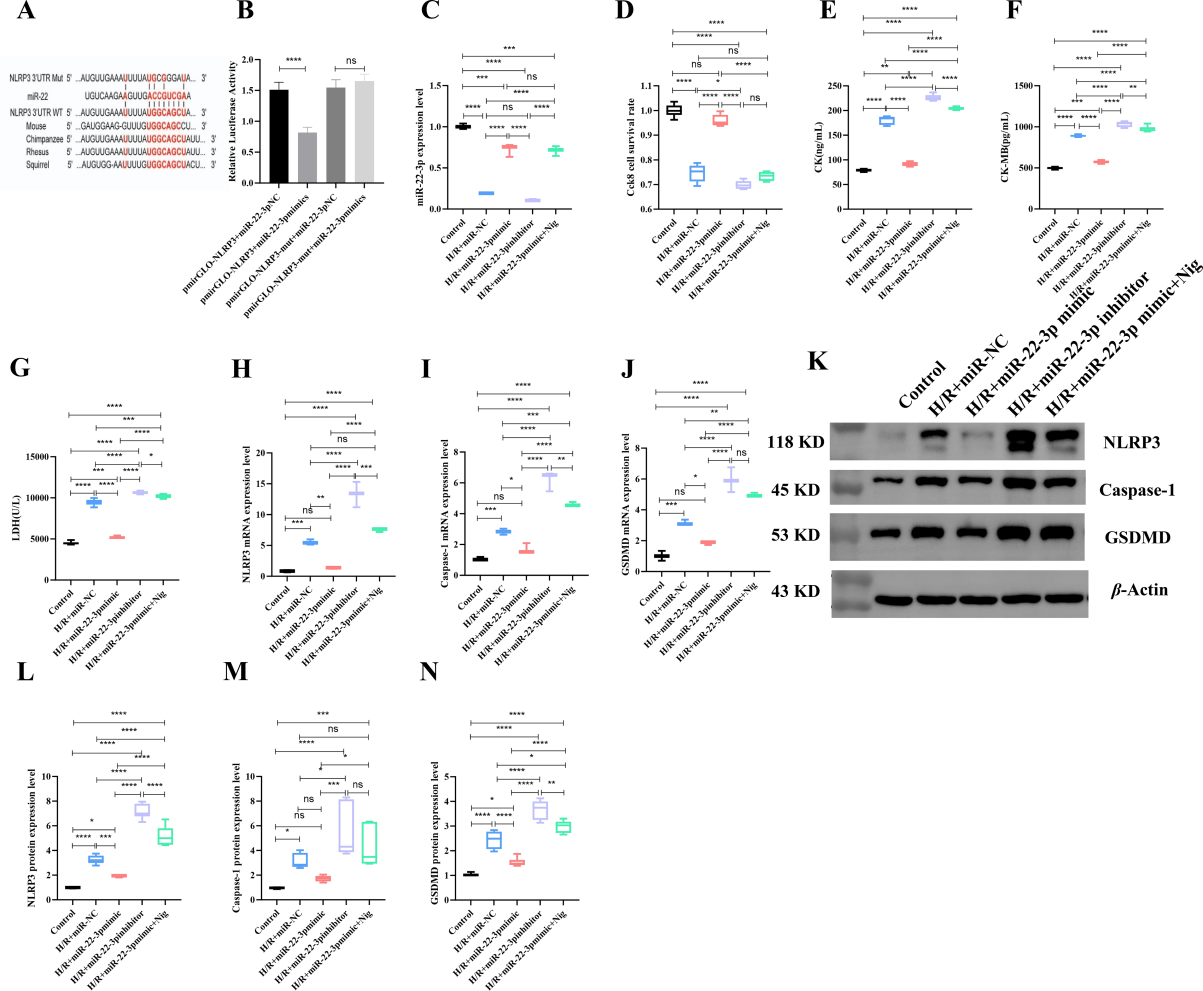
MiR-22-3p targets inhibition of NLRP3 inflammasome-mediated pyroptosis to alleviate cardiomyocyte hypoxia/reoxygenation injury. **(A)** MiRNA Target Prediction Database analysis indicated that miR-22-3p targets NLRP3. **(B)** Dual luciferase assay of miR-22-3p and NLRP3 (n = 5). **(C)** RT-qPCR analysis of miR-22-3p expression in cardiomyocytes (n = 3). **(D)** CCK-8 assay for cardiomyocyte survival rate (n = 6). **(E-G)** ELISA analysis of cardiac enzymes CK **(E)**, CK-MB **(F)**, and LDH **(G)** in cell supernatants (n = 6). **(H)** RT-qPCR for mRNA expression levels of NLRP3 **(H)**, Caspase-1 **(I)**, and GSDMD **(J)** in myocardial tissue (n = 3). **(K)** Representative immunoblotting image showing protein expression levels of NLRP3, Caspase-1, and GSDMD in myocardial tissue. **(L-N)** Western blotting analysis of NLRP3 **(L)**, Caspase-1 **(M)**, and GSDMD **(N)** protein expression levels in myocardial tissue (n = 6). ns *P* > 0.05, ^*^P < 0.05, ^**^P < 0.01, ^***^P < 0.001, ^****^P < 0.0001.

To investigate whether the potential cardiac protective effect of miR-22-3p is mediated through NLRP3, a bioinformatics analysis was conducted using the miRNA Target Prediction Database. The results suggested that NLRP3 is a potential target gene of miR-22-3p (**Figure 6A**). A dual-fluorescein luciferase reporter assay further confirmed that miR-22-3p directly binds to NLRP3, inhibiting its expression (**Figure 6B**). To verify that miR-22-3p alleviates cardiomyocyte H/R injury by targeting and inhibiting NLRP3 inflammasome-mediated pyroptosis, an H/R model was established. RT-qPCR analysis of miR-22-3p expression in cardiomyocytes revealed a significant reduction in the H/R injury group. Treatment with the miR-22-3p inhibitor further suppressed miR-22-3p expression, while treatment with miR-22-3p mimic significantly increased miR-22-3p expression in cardiomyocytes subjected to H/R injury, unaffected by the NLRP3-mediated pyroptosis inducer Nig (**Figure 6C**). As shown in **Figure 6D**, H/R injury significantly reduced cardiomyocyte viability, and the miR-22-3p inhibitor exacerbated this reduction. Treatment with the miR-22-3p mimic reversed the H/R-induced decrease in cardiomyocyte viability, but this protective effect was abolished by Nig. Additionally, ELISA analysis of cardiac enzyme levels (CK, CK-MB, and LDH) in the cell supernatant showed significantly elevated enzyme levels in the H/R injury group. Treatment with the miR-22-3p inhibitor further increased CK, CK-MB, and LDH levels. In contrast, miR-22-3p mimic significantly reduced these enzyme levels, and this effect was reversed after treatment with Nig (**Figures 6E–G**). These results suggest that miR-22-3p may significantly alleviate cardiomyocyte H/R injury by inhibiting NLRP3-mediated pyroptosis.

To verify the regulatory effect of miR-22-3p on the NLRP3-mediated pyroptosis pathway at the molecular level, RT-qPCR and WB were used to detect the expression levels of NLRP3, Caspase-1, and GSDMD in cardiomyocytes (**Figures 6H–N**). The results demonstrated that miR-22-3p effectively inhibited pyroptosis induced by H/R injury by targeting and suppressing the expression of NLRP3, thereby blocking the activation of its downstream molecules, Caspase-1 and GSDMD. However, the effect of miR-22-3p was reversed by the NLRP3-mediated pyroptosis inducer Nig. In conclusion, miR-22-3p alleviates cardiomyocyte H/R injury by targeting and inhibiting the NLRP3-mediated pyroptosis pathway.

### 3.6 Electroacupuncture alleviates MIRI by upregulating miR-22-3p to inhibit NLRP3 inflammasome-mediated pyroptosis

**Figure 7:**
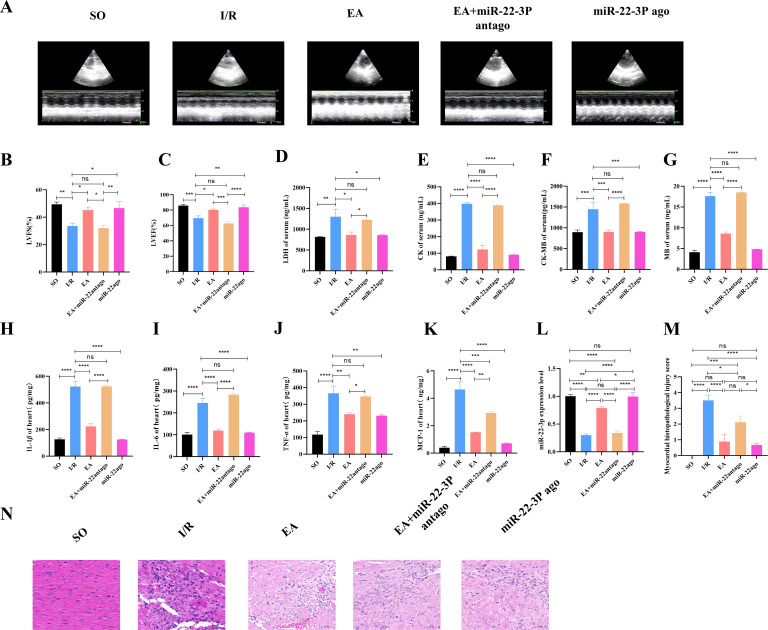
The chemical inhibition of miR-22-3p reversed the effect of electroacupuncture on mitigating MIRI damage. **(A)** Representative echocardiographic images of the left ventricle. LVFS **(B)** and LVEF **(C)** were detected by echocardiography (n = 6). Serum cardiac enzymes LDH **(D)**, CK **(E)**, CK-MB **(F)**, and MB **(G)** were analyzed by ELISA (n = 6). Myocardial inflammatory factors IL-1*β* **(H)**, IL-6 **(I)**, TNF-*α* **(J)**, and MCP-1 **(K)** were analyzed by ELISA (n = 6). **(L)** miR-22-3p expression was detected by RT-qPCR in myocardium (n = 6). **(M)** Quantitative statistics of myocardial pathological injury score (n = 6). **(N)** Hematoxylin and eosin staining to evaluate myocardial tissue pathological damage. Scale: 50 μm. ns *P* > 0.05, ^*^P < 0.05, ^**^P < 0.01, ^***^P < 0.001, ^****^P < 0.0001.

To clarify the role of miR-22-3p in EA-mediated cardioprotection, this study manipulated miR-22-3p levels in rats by tail vein injection of chemically synthesized miR-22-3p antago and miR-22-3p ago, and observed their effects on MIRI and EA efficacy. Echocardiogram results revealed that EA treatment significantly improved LVEF and LVFS after MIRI. Administration of miR-22-3p ago exhibited similar beneficial effects as EA. However, treatment with miR-22-3p antago abolished the cardioprotective effects of EA on cardiac function in MIRI model rats (**Figure 7A**). Serum cardiac enzyme profiling showed that, similar to EA treatment, miR-22-3p significantly reduced serum levels of cardiac enzymes LDH, CK, CK-MB, and Mb in MIRI model rats. After tail vein injection of miR-22-3p antago, the effect of EA treatment in reducing serum cardiac enzyme levels was abolished (**Figures 7D–G**). Additionally, EA treatment reduced the levels of inflammatory factors IL-1*β*, IL-6, TNF-*α*, and MCP-1 in ischemic myocardial tissue of MIRI model rats. Tail vein injection of miR-22-3p ago exhibited a similar effect to EA treatment, while miR-22-3p antago abolished the effect of EA in reducing inflammatory factor levels in ischemic myocardial tissue (**Figures 7H–K**). RT-qPCR analysis showed that both EA treatment and miR-22-3p ago significantly increased miR-22-3p levels in myocardial tissue of MIRI model rats, while miR-22-3p antago abolished the effect of EA treatment on elevating miR-22-3p levels (**Figure 7L**). Pathological morphological observation of myocardial tissue revealed that miR-22-3p ago had the same significant effect as EA treatment in reducing pathological injury scores, alleviating myocardial ischemia-reperfusion-induced injury, and reducing inflammatory cell infiltration. In contrast, treatment with miR-22-3p antago significantly weakened the protective effect of EA on myocardial tissue, markedly aggravating myocardial tissue edema and inflammatory infiltration (**Figures 7M, N**). These results indicate that miR-22-3p plays a pivotal role in improving cardiac function, reducing cardiac enzyme release, inhibiting inflammatory reactions, and alleviating pathological injury during EA treatment.

**Figure 8:**
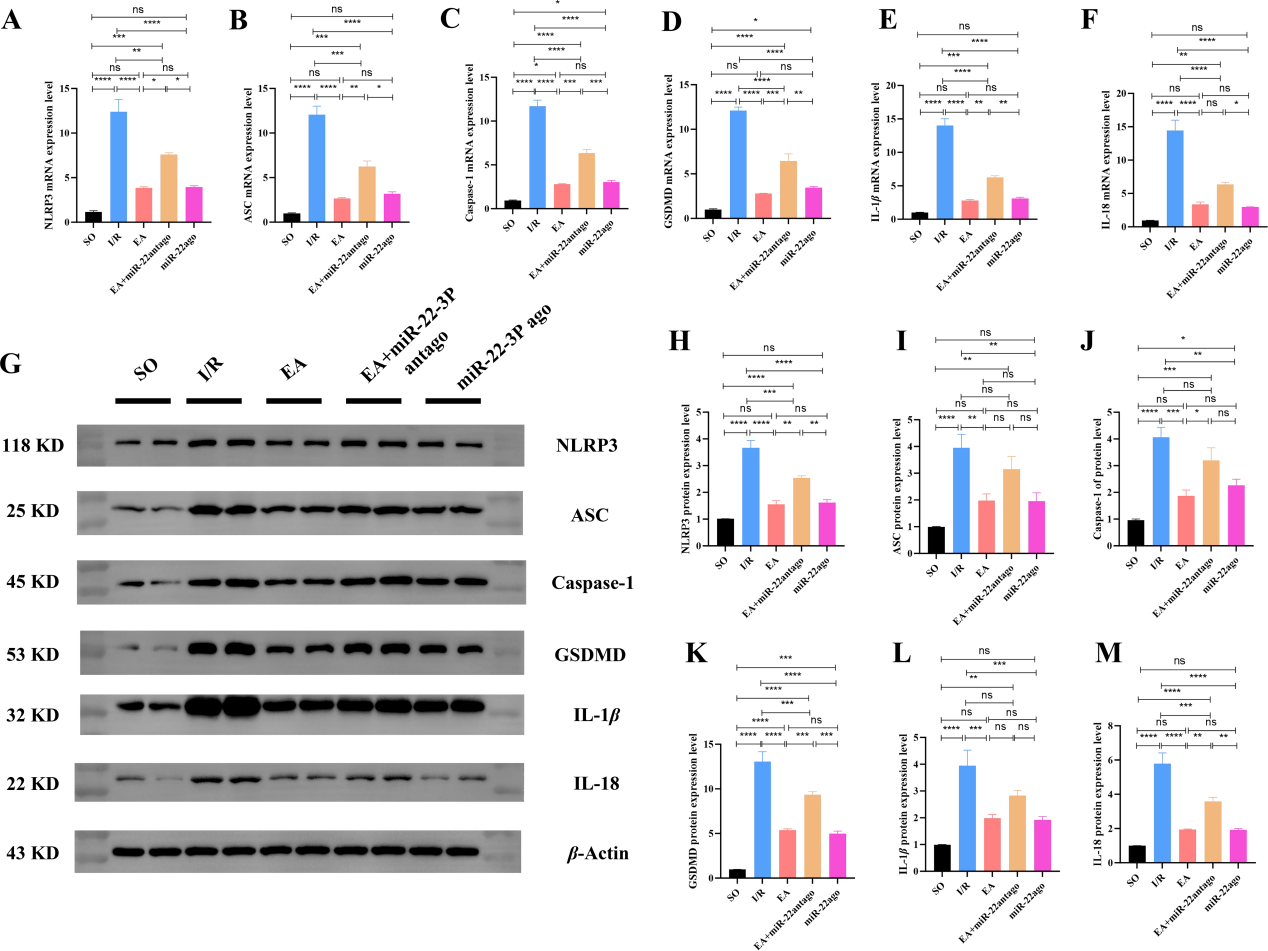
The miR-22-3p inhibitor reversed the key mRNA and protein expression of electroacupuncture, inhibiting NLRP3 inflammasome-mediated pyroptosis in cardiomyocytes. RT-qPCR was used to detect the mRNA expression levels of NLRP3 **(A)**, ASC **(B)**, Caspase-1 **(C)**, GSDMD **(D)**, IL-1*β* **(E)**, and IL-18 **(F)** in myocardial tissue (n = 3). **(G)** Representative image of protein expression levels of NLRP3, ASC, Caspase-1, GSDMD, IL-1*β*, and IL-18 in myocardial tissue by Western blotting. Western blotting was used to detect the protein expression levels of NLRP3 **(H)**, ASC **(I)**, Caspase-1 **(J)**, GSDMD **(K)**, IL-1*β* **(L)**, and IL-18 **(M)** in myocardial tissue (n = 5). ns *P* > 0.05, ^*^P < 0.05, ^**^P < 0.01, ^***^P < 0.001, ^****^P < 0.0001.

Given that miR-22-3p targets and inhibits NLRP3 inflammasome-mediated pyroptosis to alleviate cardiomyocyte H/R injury, the molecular mechanism through which EA alleviates MIRI by upregulating miR-22-3p to inhibit NLRP3 inflammasome-mediated pyroptosis was further verified *in vivo*. RT-qPCR analysis of cardiac tissue revealed that both EA treatment and miR-22-3p ago significantly suppressed the mRNA levels of NLRP3, ASC, Caspase-1, GSDMD, IL-1*β*, and IL-18. However, the use of miR-22-3p antagomir abolished the inhibitory effect of EA treatment (**Figures 8A–F**). WB results confirmed that miR-22-3p ago reduced the protein expression levels of NLRP3, ASC, Caspase-1, GSDMD, IL-1*β*, and IL-18 in ischemic cardiac muscle tissue, showing effects similar to those of EA treatment. After administration of miR-22-3p antago, EA’s inhibitory effects on the protein expression levels of these key molecules were abolished. These results confirm that EA protects against MIRI injury by upregulating serum exosome miR-22-3p levels, targeting the inhibition of NLRP3 inflammasome assembly, blocking Caspase-1/GSDMD-mediated pyroptosis, and reducing the release of inflammatory cytokines such as IL-1*β*, IL-18, IL-6, TNF-*α*, and MCP-1.

## 4 Discussion

This study presents four key findings. First, EA at acupoints exerts a specific anti-inflammatory effect to alleviate MIRI. Second, EA alleviates MIRI by inhibiting NLRP3 inflammasome-mediated pyroptosis. Third, serum exosome delivery after EA inhibits the NLRP3 inflammasome-mediated pyroptosis protective signal, providing myocardial protection in MIRI. Finally, this study identified upregulated serum exosomal miR-22-3p after EA as a key cardioprotective molecule that alleviates MIRI by inhibiting NLRP3 inflammasome-mediated pyroptosis and reducing the release of inflammatory factors.

Acupuncture and moxibustion have established clinical efficacy in alleviating ischemic heart attack. These therapies protect cardiomyocytes and alleviate MIRI by regulating autophagy, inhibiting inflammation, apoptosis, and oxidative stress. Clinical studies have shown that electrical stimulation of acupoints, such as Neiguan, reduces serum myocardial enzyme levels, mitigates inflammatory responses, improves cardiac function, and alleviates MIRI (47, 48). EA at Neiguan, Lique, and Yunmen has been shown to reduce serum myocardial cTnI levels, improve muscle strength scores, and shorten ICU stays, thereby alleviating myocardial I/R injury in adult patients undergoing heart valve replacement (49). A previous multi-center, large-sample randomized controlled clinical study confirmed that Neiguan acupuncture significantly reduced the frequency of angina attacks in patients with ischemic heart disease, alleviated the severity of these attacks, and improved quality of life (24). Clinical evaluations also demonstrated that acupuncture can enhance cardiac function by alleviating inflammatory responses, oxidative stress, and ventricular remodeling, thus safely and effectively alleviating MIRI (25). These findings support the notion that acupuncture has proven clinical efficacy in alleviating ischemic heart attack and mitigating MIRI. Current research on acupuncture’s protective effects in MIRI mainly focuses on macroscopic effect observations and verification of existing single molecular mechanisms. However, its material basis and underlying mechanistic pathways require further investigation. Acupuncture has been shown to reduce ECG ST-segment elevation, decrease myocardial infarction area, and alleviate myocardial I/R injury by inhibiting apoptosis (50). EA at PC6 significantly reduces myocardial cell apoptosis, decreases oxidative stress marker levels, enhances antioxidant enzyme (GSH-PX) activity, improves cardiac function, and effectively alleviates myocardial I/R injury (51). Acupuncture at Neiguan has also been found to reduce excessive sympathetic nerve activation, which in turn reduces the incidence and severity of arrhythmias and myocardial injury in MIRI rats (52). EA triggers the resilience of the cerebellar fastigial nucleus GABA−Glu neural pathway to late MIRI events by attenuating microglial activity (53). Previous research has shown that EA at Neiguan can directly enhance cardiac circulation (54), reduce MIRI by regulating the alternative splicing of GABRG2 *via* Nova1 expression (27), and inhibit the progression of myocardial fibrosis through suppression of MIAT expression (38). Additionally, activation of the Nrf2/HO-1 signaling pathway has been shown to inhibit cardiomyocyte ferroptosis through anti-inflammatory mechanisms (28) and protect ischemic myocardium by regulating genes involved in the NOD-like receptor signaling pathway (26). Consistent with these findings, our study demonstrated that EA can specifically improve cardiac function, reduce myocardial infarct size, lower serum myocardial enzyme levels and myocardial tissue inflammatory factors, alleviate myocardial tissue damage and inflammatory infiltration, and provide anti-inflammatory cardiac protection in MIRI rats.

As a key effector mechanism of the innate immune system, pyroptosis is triggered upon recognition of pathogenic or danger signals by pattern recognition receptors such as the NLRP3 inflammasome (55). Pyroptosis, a highly pro-inflammatory form of programmed cell death, plays a critical role in myocardial tissue damage and exacerbates local inflammation (45). This process depends on the activation of the caspase and GSDMD families, with the NLRP3/Caspase-1/GSDMD pathway being the classical mechanism mediating pyroptosis (56). Notably, the NLRP3 inflammasome functions as a PRR complex within the innate immune system, capable of sensing both PAMPs and DAMPs (57). Upon activation by ischemia-released danger signals, NLRP3 assembles into an inflammasome complex that activates caspase-1, which then cleaves GSDMD to execute pyroptosis while simultaneously maturing pro-inflammatory cytokines IL-1*β* and IL-18 (56, 57). Thus, NLRP3-mediated pyroptosis represents a critical juncture where innate immune activation drives inflammatory tissue damage. During myocardial ischemia, local risk-related molecules are released, inducing Caspase-1 activation, which further increases NLRP3 expression and promotes the release of IL-1*β* and IL-18. This cascade of events leads to inflammation, myocardial dysfunction, fibrosis, and an expanded myocardial infarction area, ultimately contributing to heart failure. Moreover, the inflammatory substances and chemokines released by myocardial pyroptosis attract immune cells, such as macrophages, to the damaged myocardium. This results in a vicious cycle of inflammation through NLRP3 inflammasome activation, further exacerbating myocardial injury, increasing the damaged area, and promoting tissue remodeling (58). By inhibiting key molecules involved in the initiation, assembly, activation, and release of inflammatory substances in the pyroptosis inflammasome, the levels of local inflammatory markers in myocardial tissue and the severity of damage in myocardial ischemia animal models can be significantly improved (59–61). This study investigated whether EA treatment alleviates MIRI by inhibiting NLRP3-mediated pyroptosis. Our results demonstrate that EA improves cardiac function, reduces myocardial enzyme release, inhibits the inflammatory response, and alleviates pathological damage by targeting the NLRP3-mediated pyroptosis pathway.

Intercellular communication plays a critical role in biological processes, with exosomes emerging as key mediators of intercellular information transfer. Exosomes, small vesicles ranging from 30 to 150 nm in diameter, are secreted by multivesicular bodies within cells and released into the extracellular matrix *via* the cell membrane (62). These vesicles carry a diverse array of bioactive substances, including nucleic acids (e.g., miRNA, messenger RNA [mRNA], long non-coding RNA [lncRNA], and tumor-derived double-stranded DNA), bioactive proteins (e.g., heat shock proteins, G proteins, membrane proteins, C-reactive lectins, growth factors, cytokines), and metabolic compounds (e.g., sugars, proteins, lipids) (63, 64). Exosomes exhibit specific targeting to regulatory elements, exerting biological effects by directly fusing with the target cell membrane to deliver miRNA into the cytoplasm, being internalized *via* endocytosis, or mediating antigen presentation or cell signaling through surface receptor interactions. Recent studies have shown that EA can deliver miRNA-381 *via* serum exosomes, reduce ECG ST segment elevation, enhance cardiac function, and lower serum cTn-I levels, thereby mitigating LPS-induced myocardial injury (36). However, it remains unclear whether serum exosomes following EA can benefit MIRI, or how local EA can achieve remote targeting of the heart to alleviate MIRI. This study demonstrates that serum exosomes after EA can be taken up by cardiomyocytes, inhibiting NLRP3 inflammasome-mediated pyroptosis to reduce H/R-induced myocardial injury, as evidenced by decreased myocardial enzyme levels in the cell supernatant, improved cell viability, and suppressed expression of pyroptosis-related mRNA and proteins. These findings suggest that EA may offer a protective effect against MIRI through the bioactive molecules carried by serum exosomes.

Current studies have demonstrated that exosomes are rich in miRNA molecules and exhibit strong selective enrichment (65, 66). Exosomal miRNAs are non-coding, single-stranded RNA molecules, approximately 20–24 nucleotides in length, encoded by endogenous genes. Their precursors are single-stranded RNAs, 70–90 bases in length, forming a hairpin structure that is processed by the endonuclease Dicer enzyme, resulting in mature miRNAs. These miRNAs regulate post-transcriptional gene expression in various body cells, influencing around one-third of human genes, and play a critical role in the intercellular communication and biological effects mediated by exosomes (67). Exosomes can transport and release miRNAs into target cells through receptor-dependent endocytosis or plasma membrane fusion. Once inside the target cells, miRNAs regulate gene expression by binding to complementary and non-complementary sites on target mRNAs (68). Serum miRNA-22 levels decrease with the progression of ischemic myocardial injury (69). It has a sensitivity of 86.00% and a specificity of 94.00% for diagnosing AMI, making it a potential biomarker for clinical diagnosis (70). Furthermore, overexpression of miRNA-22 has been shown to exert anti-inflammatory effects by inhibiting the p38MAPK/CBP/c-Jun-AP-1 signaling pathway (71), prevent myocardial cell apoptosis, and reduce the release of pro-inflammatory mediators such as TNF-*α* and IL-6 through cAMP response element binding protein expression, one of its targets, thereby protecting the myocardium (72). Additionally, miRNA-22 helps protect cardiomyocytes by regulating Sirtuin-1 and PPAR-*γ* coactivator-1*α*, thereby influencing mitochondrial oxidative stress survival and reducing peroxide levels (73). The present study reveals that EA upregulated serum exosomal miR-351-3p and miR-22-3p, while downregulating miR-1843b-5p and miR-186-5p in MIRI model rats. In our clinical trial with AMI-PCI patients, EA upregulated 61 key serum exosomal miRNAs and downregulated 48 key serum exosomal miRNAs. Based on these findings, miR-22-3p was identified as a key serum exosomal miRNA contributing to the reduction of MIRI by EA. Bioinformatics and quantitative analyses further indicated that NLRP3 is an anti-inflammatory target of miR-22-3p in cardiomyocytes. The study confirmed that miR-22-3p significantly inhibited pyroptosis in cardiomyocytes induced by ischemia-reperfusion and suppressed the expression of NLRP3 inflammasome-mediated pyroptosis markers, including NLRP3, ASC, Caspase1, and GSDMD. The release of inflammatory cytokines such as IL-1*β*, IL-18, IL-6, TNF-*α*, and MCP-1 was also reduced. Moreover, heart-specific inhibition of miR-22-3p *in vivo* diminished the cardioprotective effects of EA and increased the expression of NLRP3, ASC, Caspase1, and GSDMD in the heart, suggesting that miR-22-3p mitigates pyroptosis by inhibiting NLRP3 inflammasome-mediated key molecules in the ischemic/reperfusion heart, thus playing an anti-inflammatory role in myocardial protection.

Previous studies have demonstrated that exosomes and their carried miRNAs play a critical role in mediating acupuncture effects (74, 75). Detecting exosomes in interstitial fluid during acupuncture intervention enables real-time monitoring of patient responses and mechanistic exploration of acupuncture (76). Electrically stimulated acupuncture elevates serum exosomal miR-181 levels to enhance renal blood perfusion (77). It also downregulates serum exosomal let-7-5p to activate the Igf1 signaling axis, boost skeletal muscle protein synthesis, and prevent muscular atrophy (78). In middle cerebral artery occlusion rat models, EA upregulates serum exosomal miR-210 to activate the HIF-1*α*/VEGF/Notch1 signaling pathway, thereby facilitating post-stroke angiogenesis (79). Moreover, EA increases exosomal miR-146b expression to promote the differentiation of endogenous neural stem cells in the peri-ischemic striatum and alleviate neurological damage following ischemic stroke (80). In rat models of sciatic nerve injury, EA facilitates nerve regeneration and functional recovery of damaged sciatic nerves via exosome-mediated delivery of miR-21 (76). EA improves intestinal motility in slow-transit constipation by upregulating serum exosomal miR-34c-5p and activating the SCF/c-Kit signaling pathway (81). Notably, in emphysema mouse models, EA markedly mitigates pulmonary inflammatory responses by suppressing NLRP3 inflammasome activation and the release of inflammation-induced detrimental exosomes (82). Additionally, EA attenuates LPS-induced myocardial injury through serum exosome-delivered miR-381 (36). EA preconditioning serves as a vital cardioprotective strategy (83–90). Accumulating evidence has confirmed that EA preconditioning inhibits NLRP3 inflammasome activation and relieves acute myocardial ischemic injury as well as MIRI (91, 92). Mechanistically, existing research has validated the pivotal function of exosomes and exosomal miRNAs in EA-induced therapeutic effects. Specifically, EA restrains NLRP3 inflammasome activation and the secretion of pro-inflammatory harmful exosomes to ameliorate pulmonary inflammation. In ischemic heart diseases, EA preconditioning has garnered extensive attention for its promising therapeutic potential, as it suppresses NLRP3 inflammasome activation and reduces myocardial damage. For the first time, the present study verified through both animal experiments and clinical observations that following MIRI, EA specifically increases serum exosomal miR-22-3p. By directly targeting NLRP3, miR-22-3p blocks the NLRP3/ASC/Caspase-1/GSDMD signaling cascade, inhibits cardiomyocyte pyroptosis, and ultimately alleviates MIRI. First, these findings provide solid scientific evidence for the anti-pyroptosis and anti-inflammatory mechanisms of EA in the treatment of cardiac diseases. Second, *in vitro* cellular experiments confirmed that serum exosomes and miR-22-3p derived from EA intervention exert cardioprotective effects via pyroptosis inhibition, indicating promising potential for clinical translation and application. Third, from an immunological perspective, this study clarified the direct targeting relationship between miR-22-3p and NLRP3 in the context of MIRI, establishing an intrinsic linkage among the cardioprotective effects of EA, non-coding RNAs, and innate immune signaling pathways.

This study has several limitations. First, while our data suggest that EA upregulates the serum exosomal miR-22-3p level and targets NLRP3 inflammasome-mediated pyroptosis, further investigation is required to fully elucidate the origin of serum exosomal miR-22-3p. Second, it was not feasible to manipulate the loss of function of circulating exosomes in the context of the cardioprotective effects of EA, as no animal model or *in vivo* pharmacological inhibitors have been developed to block exosome production. Our analysis was limited to evaluating the effects and mechanisms of serum exosomes post-EA in a myocardial H/R model *in vitro*. Specifically, although this study has effectively confirmed that Exo-EA can deliver miR-22-3p and inhibit pyroptosis *in vitro*, there is a lack of *in vivo* experimental evidence to prove that circulating exosomes are the key mediator of the cardioprotective effect of EA. We failed to supplement intervention experiments with exosome production inhibitors (such as GW4869) or compare the effect of EA before and after serum exosome depletion, which limits the confirmation of the key role of circulating exosomes in the cardioprotective effect of EA. Third, regarding clinical research, the sample size of the clinical cohort in this study is small (only 10 cases in each group), which leads to insufficient statistical power for small RNA sequencing. Although we have screened miR-22-3p as the core target, there is a lack of verification experiments in an independent clinical cohort, which restricts the reliability of the research conclusions. Fourth, while miRNAs are known for their broad regulatory capabilities, and existing data highlight miR-22-3p as a key mediator in EA-induced inhibition of NLRP3 inflammasome-mediated pyroptosis to protect the heart, the specific mechanisms of other downstream signaling pathways remain to be explored. Finally, despite the wealth of serum exosomal small RNA sequencing data from both experimental animals and clinical subjects, the role of other potential key exosomal miRNAs in acupuncture’s effects on MIRI has yet to be experimentally validated.

**Figure 9:**
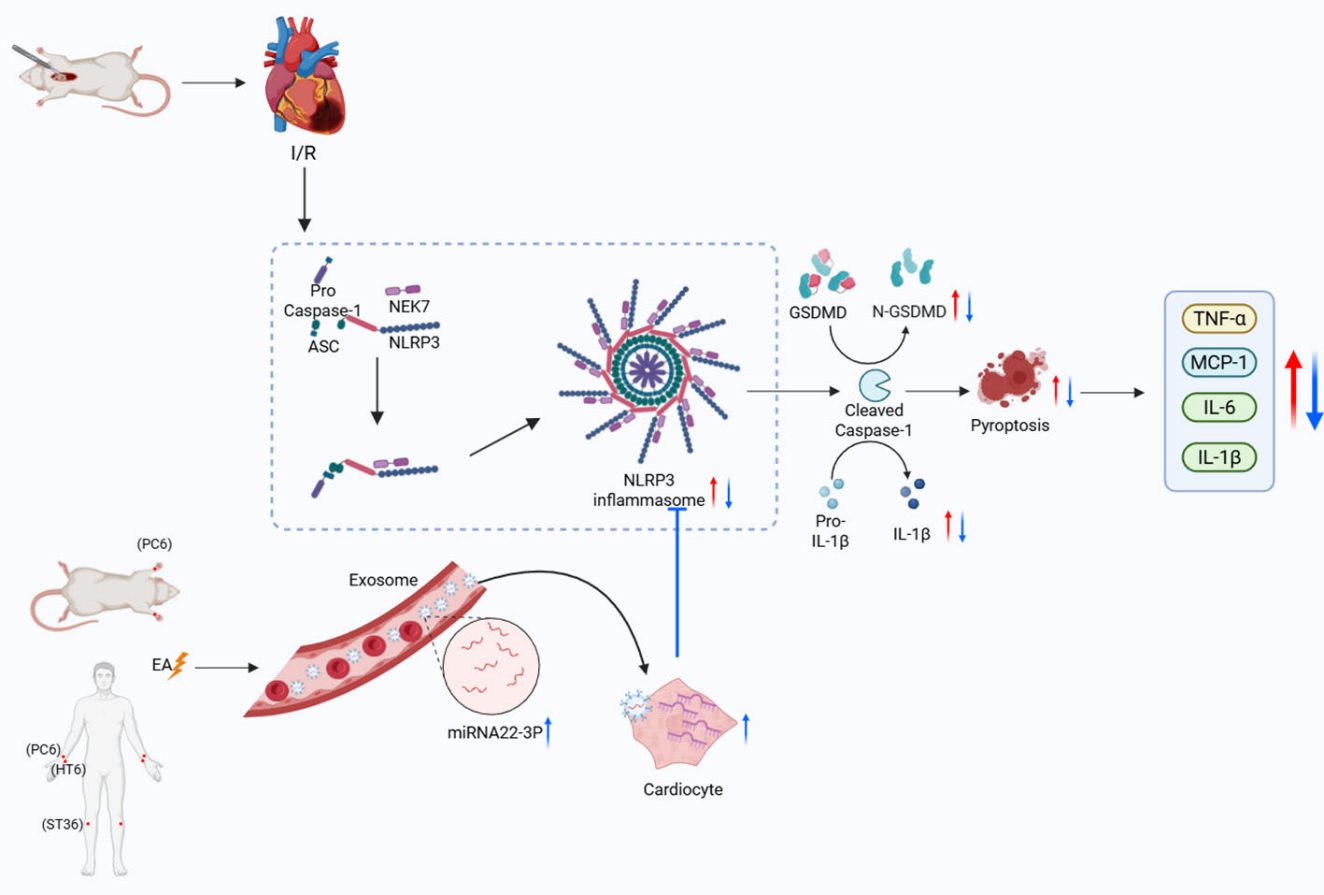
The mechanism by which electroacupuncture post-treatment serum exosomes exert anti-inflammatory effects to protect the heart and alleviate MIRI. Electroacupuncture confers cardioprotective effects by upregulating the level of miR-22-3p in serum exosomes, which are carried and delivered to the heart through exosomes. These miRNAs target and inhibit NLRP3 inflammasome-mediated apoptosis signals, thereby alleviating the inflammatory response and mitigating MIRI. Red arrows represent disease-induced changes; green arrows represent EA-induced changes (Created with BioRender).

In conclusion, EA provides targeted cardiac protection against inflammation and alleviates MIRI. Our findings confirm that EA improves cardiac function, reduces myocardial enzyme release, inhibits the inflammatory response, and mitigates pathological damage by inhibiting the NLRP3-mediated pyroptosis pathway. Serum exosomes following EA play a pivotal role in the cardiac protective mechanism of MIRI through pyroptosis-mediated anti-inflammatory effects. miR-22-3p was identified as the central molecule in EA’s cardioprotective mechanism, reducing the release of inflammatory factors (IL-1*β*, IL-18, IL-6, TNF-*α*, and MCP-1) by inhibiting NLRP3 inflammasome-mediated pyroptosis. Inhibition of miR-22-3p abolished EA’s cardioprotective effects. Our findings reveal a novel mechanism by which EA alleviates MIRI by upregulating serum exosomal miR-22-3p, targeting the inhibition of NLRP3 inflammasome-mediated pyroptosis, and reducing the release of inflammatory cytokines (IL-1*β*, IL-18, IL-6, TNF-*α*, and MCP-1) (**Figure 9**).

## Data Availability

The datasets used or analyzed in this study are available from the corresponding author upon reasonable request. The small RNA sequencing data of rat serum exosomes have been successfully uploaded and assigned the accession number CRA040320 (available at: https://ngdc.cncb.ac.cn/gsa/s/dU2ZzPZx). The small RNA sequencing data of Serum exosomes from the enrolled patients have been successfully uploaded and assigned the accession number HRA017517 (available at: https://ngdc.cncb.ac.cn/gsa-human/s/h5OGuld8).

## Glossary

AMI: Acute myocardial infarction
CCK-8: Cell Counting Kit-8
Caspase-1: Cysteine aspartic acid-specific protease-1
CK: Creatine kinase
CK-MB: Creatine kinase-MB
ECG: Electrocardiography
EA group: Electroacupuncture at acupoints group
ELISA: Enzyme-Linked Immunosorbent Assay
Exo: Exosome
FS: Left ventricular fraction shortening
GSDMD: Gasdermin D
H/R: Hypoxia/Reoxygenation
HE: Hematoxylin-Eosin staining
I/R group: Myocardial ischemia-reperfusion injury group;;IL-1β, Interleukin-1β
IL-6: Interleukin-6
IL-18: Interleukin-18
LDH: Lactate dehydrogenase
LVEF: Left ventricular ejection fraction
Mb: Myoglobin
MCP-1: Monocyte chemoattractant protein-1
miRNA: MicroRNA
mRNA: Messenger RNA
MIRI: Myocardial ischemia–reperfusion injury
NA group: Electroacupuncture at non-meridian and non-acupoint group
Nig: Nigericin
NLRP3: NOD-like receptor pyrin domain-containing protein 3
PCI: Percutaneous coronary intervention
SA group: Electroacupuncture at sham acupoint group
SO group: Sham operation group
TNF-α: Tumor Necrosis Factor-α
RT-qPCR: Reverse Transcription Quantitative Polymerase Chain Reaction
WB: Western Blotting

## References

[1] GBD 2021 Causes of Death Collaborators . Global burden of 288 causes of death and life expectancy decomposition in 204 countries and territories and 811 subnational locations, 1990-2021: a systematic analysis for the Global Burden of Disease Study 2021. Lancet. (2024) 403:2100–32. doi: 10.1016/S0140-6736(24)00367-2 PMC11126520 38582094

[2] Byrne RA Rossello X Coughlan JJ Barbato E Berry C Chieffo A . 2023 ESC Guidelines for the management of acute coronary syndromes. Eur Heart J. (2023) 44:3720–826. doi: 10.1093/ehjacc/zuad107. PMID: 37622654

[3] Li Y Li Y Li Y Jiang Y Wang M Wang M . MTX2 facilitates PKM2 tetramerization to promote cardiac glucose metabolism and protects the heart against ischemia/reperfusion injury. Theranostics. (2025) 15:6737–52. doi: 10.7150/thno.110162. PMID: 40585998 PMC12203670

[4] Peng Y Shi X Wang Q Pan Y Wang X Yan K . Synthesis and biological evaluation of Sulforaphane derivatives with dual functions: Ischemia-reperfusion injury protection and antitumor effects. Bioorg Med Chem. (2025) 129:118298. doi: 10.1016/j.bmc.2025.118298. PMID: 40633511

[5] Xiang Q Yi X Zhu X-H Wei X Jiang D-S . Regulated cell death in myocardial ischemia-reperfusion injury. Trends Endocrinol Metabolism: TEM. (2024) 35:219–34. doi: 10.1016/j.tem.2023.10.010. PMID: 37981501

[6] Peng Y Tao Y Liu L Zhang J Wei B . Crosstalk among reactive oxygen species, autophagy and metabolism in myocardial ischemia and reperfusion stages. Aging Dis. (2024) 15:1075–107. doi: 10.14336/ad.2023.0823-4. PMID: 37728583 PMC11081167

[7] Du B Fu Q Yang Q Yang Y Li R Yang X . Different types of cell death and their interactions in myocardial ischemia-reperfusion injury. Cell Death Discov. (2025) 11:87. doi: 10.1038/s41420-025-02372-5. PMID: 40044643 PMC11883039

[8] Pandolfi S Valdenassi L Franzini M Simonetti V Chirumbolo S . Insights into the use of oxygen-ozone therapy in ischemic cardiopathy and cardiovascular disease: a role for mitochondria? Med Gas Res. (2024) 14:164–6. doi: 10.4103/mgr.medgasres-d-23-00007. PMID: 40434379 PMC11257178

[9] Pandolfi S Chirumbolo S Franzini M Tirelli U Valdenassi L . Oxygen-ozone therapy for myocardial ischemic stroke and cardiovascular disorders. Med Gas Res. (2025) 15:36–43. doi: 10.4103/mgr.medgasres-d-23-00013. PMID: 39217427 PMC11515079

[10] Bertero E Popoiu TA Maack C . Mitochondrial calcium in cardiac ischemia/reperfusion injury and cardioprotection. Basic Res Cardiol. (2024) 119:569–85. doi: 10.1007/s00395-024-01060-2. PMID: 38890208 PMC11319510

[11] Toldo S Abbate A . The NLRP3 inflammasome in acute myocardial infarction. Nat Rev Cardiol. (2018) 15:203–14. doi: 10.1038/nrcardio.2017.161. PMID: 29143812

[12] Seoane PI Lee B Hoyle C Yu S Lopez-Castejon G Lowe M . The NLRP3-inflammasome as a sensor of organelle dysfunction. J Cell Biol. (2020) 219:e202006194. doi: 10.1083/jcb.202006194. PMID: 33044555 PMC7543090

[13] Shi J Zhao Y Wang K Shi X Wang Y Huang H . Cleavage of GSDMD by inflammatory caspases determines pyroptotic cell death. Nature. (2015) 526:660–5. doi: 10.1038/nature15514. PMID: 26375003

[14] Yu P Zhang X Liu N Tang L Peng C Chen X . Pyroptosis: mechanisms and diseases. Signal Transduction Targeted Ther. (2021) 6:128. doi: 10.1038/s41392-021-00507-5. PMID: 33776057 PMC8005494

[15] Pinzón-Fernández MV Saavedra-Torres JS López Garzón NA Pachon-Bueno JS Tamayo-Giraldo FJ Rojas Gomez MC . NLRP3 and beyond: inflammasomes as central cellular hub and emerging therapeutic target in inflammation and disease. Front Immunol. (2025) 16:1624770. doi: 10.3389/fimmu.2025.1624770 40959087 PMC12433879

[16] Man SM Kanneganti TD . Innate immune sensing of cell death in disease and therapeutics. Nat Cell Biol. (2024) 26:1420–33. doi: 10.1038/s41556-024-01491-y. PMID: 39223376 PMC12459733

[17] Kumar R Singh A Tripathi S Salahuddin N Abdullah MM . Targeting the NLRP3 inflammasome with natural products for ischemia-reperfusion injury across organs: mechanisms, structure-activity relationships, and delivery innovations. Inflammopharmacology. (2025) 33:6589–634. doi: 10.1007/s10787-025-02007-2. PMID: 41105346

[18] Toldo S Mauro AG Cutter Z Abbate A . Inflammasome, pyroptosis, and cytokines in myocardial ischemia-reperfusion injury. Am J Physiol Heart Circ Physiol. (2018) 315:H1553–68. doi: 10.1152/ajpheart.00158.2018. PMID: 30168729 PMC6336966

[19] Zhaolin Z Guohua L Shiyuan W Zuo W . Role of pyroptosis in cardiovascular disease. Cell Prolif. (2019) 52:e12563. doi: 10.1111/cpr.12563. PMID: 30525268 PMC6496801

[20] Zhang J Huang L Shi X Yang L Hua F Ma J . Metformin protects against myocardial ischemia-reperfusion injury and cell pyroptosis via AMPK/NLRP3 inflammasome pathway. Aging. (2020) 12(23):24270–87. doi: 10.18632/aging.202143 PMC7762510 33232283

[21] Liu S Bi Y Han T Li YE Wang Q Wu NN . The E3 ubiquitin ligase MARCH2 protects against myocardial ischemia-reperfusion injury through inhibiting pyroptosis via negative regulation of PGAM5/MAVS/NLRP3 axis. Cell Discov. (2024) 10:24. doi: 10.1038/s41421-023-00622-3. PMID: 38409220 PMC10897310

[22] Swanson KV Deng M Ting JPY . The NLRP3 inflammasome: molecular activation and regulation to therapeutics. Nat Rev Immunol. (2019) 19:477–89. doi: 10.1038/s41577-019-0165-0. PMID: 31036962 PMC7807242

[23] Lu L Zhang Y Tang X Ge S Wen H Zeng J . Evidence on acupuncture therapies is underused in clinical practice and health policy. BMJ. (2022) 376:e067475. doi: 10.1136/bmj-2021-067475. PMID: 35217525 PMC8868048

[24] Zhao L Li D Zheng H Chang X Cui J Wang R . Acupuncture as adjunctive therapy for chronic stable angina: a randomized clinical trial. JAMA Intern Med. (2019) 179:1388–97. doi: 10.1001/jamainternmed.2019.2407. PMID: 31355870 PMC6664382

[25] Xiong J Wei Y Huang X Hu J Ling F Shang Z . A meta-analysis of randomized controlled trials (RCTs) investigating the efficacy and safety of acupuncture in treating myocardial ischemia/reperfusion (I/R) injury. Cardiol Res Pract. (2025) 2025:9970541. doi: 10.1155/crp/9970541. PMID: 40599673 PMC12213044

[26] Huang Y Lu S-F Hu C-J Fu S-P Shen W-X Liu W-X . Electro-acupuncture at Neiguan pretreatment alters genome-wide gene expressions and protects rat myocardium against ischemia-reperfusion. Molecules. (2014) 19:16158–78. doi: 10.3390/molecules191016158. PMID: 25302705 PMC6271995

[27] Qi W Fu H Luo X Ren Y Liu X Dai H . Electroacupuncture at PC6 (Neiguan) attenuates angina pectoris in rats with myocardial ischemia-reperfusion injury through regulating the alternative splicing of the major inhibitory neurotransmitter receptor GABRG2. J Cardiovasc Transl Res. (2022) 15:1176–91. doi: 10.1007/s12265-022-10245-w. PMID: 35377129

[28] Li X Sun Y-X Tjahjono AW Wei Y Li X Zheng Q-H . Acupuncture attenuates myocardial ischemia/reperfusion injury-induced ferroptosis via the Nrf2/HO-1 pathway. Chin Med. (2025) 20:61. doi: 10.1186/s13020-025-01114-0. PMID: 40346679 PMC12065278

[29] Sahoo S Adamiak M Mathiyalagan P Kenneweg F Kafert-Kasting S Thum T . Therapeutic and diagnostic translation of extracellular vesicles in cardiovascular diseases: roadmap to the clinic. Circulation. (2021) 143(14):1426–49. doi: 10.1161/CIRCULATIONAHA.120.049254 PMC8021236 33819075

[30] Ma D Guan B Song L Liu Q Fan Y Zhao L . A bibliometric analysis of exosomes in cardiovascular diseases from 2001 to 2021. Front Cardiovasc Med. (2021) 8:734514. doi: 10.3389/fcvm.2021.734514. PMID: 34513962 PMC8424118

[31] Liu Q Piao H Wang Y Zheng D Wang W . Circulating exosomes in cardiovascular disease: novel carriers of biological information. Biomedicine Pharmacotherapy = Biomedecine Pharmacotherapie. (2021) 135:111148. doi: 10.1016/j.biopha.2020.111148. PMID: 33412387

[32] Zheng D Huo M Li B Wang W Piao H Wang Y . The role of exosomes and exosomal microRNA in cardiovascular disease. Front Cell Dev Biol. (2020) 8:616161. doi: 10.3389/fcell.2020.616161. PMID: 33511124 PMC7835482

[33] Liu Z Zhu D Yu F Yang M Huang D Ji Z . Exosomal miR-17-3p alleviates programmed necrosis in cardiac ischemia/reperfusion injury by regulating TIMP3 expression. Oxid Med Cell Longevity. (2022) 2022:2785113. doi: 10.1155/2022/2785113. PMID: 35116091 PMC8807034

[34] Li D Zhao Y Zhang C Wang F Zhou Y Jin S . Plasma exosomes at the late phase of remote ischemic pre-conditioning attenuate myocardial ischemia-reperfusion injury through transferring miR-126a-3p. Front Cardiovasc Med. (2021) 8:736226. doi: 10.3389/fcvm.2021.736226. PMID: 34917657 PMC8669347

[35] Hou Z Qin X Hu Y Zhang X Li G Wu J . Longterm exercise-derived exosomal miR-342-5p: a novel exerkine for cardioprotection. Circ Res. (2019) 124:1386–400. doi: 10.1161/CIRCRESAHA.118.314635 30879399

[36] Chen Y Chen S Zhang J Hu X Li N Liu Z . Electroacupuncture pre-treatment exerts a protective effect on LPS-induced cardiomyopathy in mice through the delivery of miR-381 via exosomes. Biochim Biophys Acta Mol Basis Dis. (2024) 1870:167208. doi: 10.1016/j.bbadis.2024.167208. PMID: 38701956

[37] Zhao Y Ling F Qin Y Xie W Qi W Nie Q . The effect and safety of acupuncture as adjunctive therapy for STEMI patients after PCI: study protocol of a randomized controlled trial. BMC Complementary Med Ther. (2024) 24:306. doi: 10.1186/s12906-024-04608-w. PMID: 39143484 PMC11325820

[38] Qi W Li X Ren Y Liu X Fu H Wang X . Downregulation of lncRNA Miat contributes to the protective effect of electroacupuncture against myocardial fibrosis. Chin Med. (2022) 17:57. doi: 10.1186/s13020-022-00615-6. PMID: 35578250 PMC9112552

[39] Théry C Witwer KW Aikawa E Alcaraz MJ Anderson JD Andriantsitohaina R . Minimal information for studies of extracellular vesicles 2018 (MISEV2018): a position statement of the International Society for Extracellular Vesicles and update of the MISEV2014 guidelines. J Extracell Vesicles. (2018) 7:1535750. doi: 10.1080/20013078.2018.1535750 30637094 PMC6322352

[40] Robinson MD McCarthy DJ Smyth GK . edgeR: a bioconductor package for differential expression analysis of digital gene expression data. Bioinformatics. (2010) 26:139–40. doi: 10.1093/bioinformatics/btp616. PMID: 19910308 PMC2796818

[41] Anders S Huber W . Differential expression analysis for sequence count data. Genome Biol. (2010) 11:R106. doi: 10.1038/npre.2010.4282.2. PMID: 20979621 PMC3218662

[42] Li J Xu P Hong Y Xie Y Peng M Sun R . Lipocalin-2-mediated astrocyte pyroptosis promotes neuroinflammatory injury via NLRP3 inflammasome activation in cerebral ischemia/reperfusion injury. J Neuroinflamm. (2023) 20:148. doi: 10.1186/s12974-023-02819-5. PMID: 37353794 PMC10288712

[43] Xiong J Li X Fu H Luo X Li X Ren Y . Study on the specific expression of infrared radiation temperature on the body surface of acupoint in rats with chronic myocardial ischemic injury. Curr Med Imaging. (2023) 19:1580–90. doi: 10.2174/1573405619666230217120343. PMID: 36799419

[44] Oh C Spears TJ Aachoui Y . Inflammasome-mediated pyroptosis in defense against pathogenic bacteria. Immunol Rev. (2025) 329:e13408. doi: 10.1111/imr.13408. PMID: 39404258 PMC11741929

[45] Jia C Chen H Zhang J Zhou K Zhuge Y Niu C . Role of pyroptosis in cardiovascular diseases. Int Immunopharmacol. (2019) 67:311–8. doi: 10.1016/j.intimp.2018.12.028. PMID: 30572256

[46] Li X Zhang Z Han Y Zhang M . NLRP3 inflammasome and pyroptosis: implications in inflammation and multisystem disorders. PeerJ. (2025) 13:e19887. doi: 10.7717/peerj.19887. PMID: 40832584 PMC12360325

[47] Ni X Xie Y Wang Q Zhong H Chen M Wang F . Cardioprotective effect of transcutaneous electric acupoint stimulation in the pediatric cardiac patients: a randomized controlled clinical trial. Paediatric Anaesthesia. (2012) 22:805–11. doi: 10.1111/j.1460-9592.2012.03822.x. PMID: 22380768

[48] Wang Q Liang D Wang F Li W Han Y Zhang W . Efficacy of electroacupuncture pretreatment for myocardial injury in patients undergoing percutaneous coronary intervention: a randomized clinical trial with a 2-year follow-up. Int J Cardiol. (2015) 194:28–35. doi: 10.1016/j.ijcard.2015.05.043. PMID: 26011261

[49] Yang L Yang J Wang Q Chen M Lu Z Chen S . Cardioprotective effects of electroacupuncture pretreatment on patients undergoing heart valve replacement surgery: a randomized controlled trial. Ann Thorac Surg. (2010) 89:781–6. doi: 10.1016/j.athoracsur.2009.12.003. PMID: 20172127

[50] Gao J Fu W Jin Z Yu X . Acupuncture pretreatment protects heart from injury in rats with myocardial ischemia and reperfusion via inhibition of the beta(1)-adrenoceptor signaling pathway. Life Sci. (2007) 80:1484–9. doi: 10.1016/j.lfs.2007.01.019. PMID: 17303176

[51] Zhang H Liu L Huang G Zhou L Wu W Zhang T . Protective effect of electroacupuncture at the Neiguan point in a rabbit model of myocardial ischemia-reperfusion injury. Can J Cardiol. (2009) 25:359–63. doi: 10.1016/s0828-282x(09)70095-9. PMID: 19536377 PMC2722479

[52] Qianhui S Kai C Xingye D Zhiwen Y Xiaoling WU Chang XU . Effect of electroacupuncture at Neiguan (PC6) at different time points on myocardial ischemia reperfusion arrhythmia in rats. J Tradit Chin Med. (2024) 44:113–21. doi: 10.19852/j.cnki.jtcm.20231110.004 PMC10774726 38213246

[53] Zhang F Wang Q-Y Zhou J Zhou X Wei X Hu L . Electroacupuncture attenuates myocardial ischemia-reperfusion injury by inhibiting microglial engulfment of dendritic spines. iScience. (2023) 26:107645. doi: 10.1016/j.isci.2023.107645. PMID: 37670780 PMC10475514

[54] Zhuang Y Zhou J Zhou Y-M Chen J Wu P Lyu P-R . Influence of acupuncture on microcirculation perfusion of pericardium meridian and heart in acute myocardial ischemia model rats. Chin J Integr Med. (2022) 28:69–75. doi: 10.1007/s11655-021-3294-9. PMID: 34816366

[55] Nozaki K Li L Miao EA . Innate sensors trigger regulated cell death to combat intracellular infection. Annu Rev Immunol. (2022) 40:469–98. doi: 10.1146/annurev-immunol-101320-011235. PMID: 35138947 PMC9614550

[56] Shi J Gao W Shao F . Pyroptosis: Gasdermin-mediated programmed necrotic cell death. Trends Biochem Sci. (2017) 42:245–54. doi: 10.1016/j.tibs.2016.10.004. PMID: 27932073

[57] Wang Y Liu X Shi H Yu Y Yu Y Li M . NLRP3 inflammasome, an immune-inflammatory target in pathogenesis and treatment of cardiovascular diseases. Clin Transl Med. (2020) 10:91–106. doi: 10.1002/ctm2.13. PMID: 32508013 PMC7240865

[58] Liu Y Li X Sun T Li T Li Q . Pyroptosis in myocardial ischemia/reperfusion and its therapeutic implications. Eur J Pharmacol. (2024) 971:176464. doi: 10.1016/j.ejphar.2024.176464. PMID: 38461908

[59] Ding H-S Huang Y Qu J-F Wang Y-J Huang Z-Y Wang F-Y . Panaxynol ameliorates cardiac ischemia/reperfusion injury by suppressing NLRP3-induced pyroptosis and apoptosis via HMGB1/TLR4/NF-κB axis. Int Immunopharmacol. (2023) 121:110222. doi: 10.1016/j.intimp.2023.110222. PMID: 37343367

[60] Gastaldi S Giordano M Blua F Rubeo C Boscaro V Femminò S . Novel NLRP3 inhibitor INF195: Low doses provide effective protection against myocardial ischemia/reperfusion injury. VascPharmacol. (2024) 156:107397. doi: 10.1016/j.vph.2024.107397. PMID: 38897555

[61] Toldo S Mauro AG Cutter Z Van Tassell BW Mezzaroma E Del Buono MG . The NLRP3 inflammasome inhibitor, OLT1177 (Dapansutrile), reduces infarct size and preserves contractile function after ischemia reperfusion injury in the mouse. J Cardiovasc Pharmacol. (2019) 73:215–22. doi: 10.1097/fjc.0000000000000658. PMID: 30747785

[62] Meldolesi J . Exosomes and ectosomes in intercellular communication. Curr Biology: CB. (2018) 28:R435–44. doi: 10.1016/j.cub.2018.01.059. PMID: 29689228

[63] Corrado C Raimondo S Chiesi A Ciccia F De Leo G Alessandro R . Exosomes as intercellular signaling organelles involved in health and disease: basic science and clinical applications. Int J Mol Sci. (2013) 14:5338–66. doi: 10.3390/ijms14035338. PMID: 23466882 PMC3634447

[64] Malik ZA Kott KS Poe AJ Kuo T Chen L Ferrara KW . Cardiac myocyte exosomes: stability, HSP60, and proteomics. Am J Physiol Heart Circ Physiol. (2013) 304:H954–965. doi: 10.1152/ajpheart.00835.2012. PMID: 23376832 PMC3625894

[65] Xie S Zhang Q Jiang L . Current knowledge on exosome biogenesis, cargo-sorting mechanism and therapeutic implications. Membranes. (2022) 12:498. doi: 10.3390/membranes12050498. PMID: 35629824 PMC9144303

[66] Garcia-Martin R Wang G Brandão BB Zanotto TM Shah S Kumar Patel S . MicroRNA sequence codes for small extracellular vesicle release and cellular retention. Nature. (2022) 601:446–51. doi: 10.1038/s41586-021-04234-3. PMID: 34937935 PMC9035265

[67] Li C Ni Y-Q Xu H Xiang Q-Y Zhao Y Zhan J-K . Roles and mechanisms of exosomal non-coding RNAs in human health and diseases. Signal Transduction Targeted Ther. (2021) 6:383. doi: 10.1038/s41392-021-00779-x. PMID: 34753929 PMC8578673

[68] Dilsiz N . Role of exosomes and exosomal microRNAs in cancer. Future Sci OA. (2020) 6:FSO465. doi: 10.2144/fsoa-2019-0116. PMID: 32257377 PMC7117563

[69] Tu Y Wan L Zhao D Bu L Dong D Yin Z . *In vitro* and *in vivo* direct monitoring of miRNA-22 expression in isoproterenol-induced cardiac hypertrophy by bioluminescence imaging. Eur J Nucl Med Mol Imaging. (2014) 41:972–84. doi: 10.1007/s00259-013-2596-3 24504502

[70] Wang X Tian L Sun Q . Diagnostic and prognostic value of circulating miRNA-499 and miRNA-22 in acute myocardial infarction. J Clin Lab Anal. (2020) 34:2410–7. doi: 10.1002/jcla.23332. PMID: 32529742 PMC7439427

[71] Yang J Fan Z Yang J Ding J Yang C Chen L . microRNA-22 attenuates myocardial ischemia-reperfusion injury via an anti-inflammatory mechanism in rats. Exp Ther Med. (2016) 12:3249–55. doi: 10.3892/etm.2016.3777. PMID: 27882145 PMC5103773

[72] Yang J Chen L Yang J Ding J Li S Wu H . MicroRNA-22 targeting CBP protects against myocardial ischemia-reperfusion injury through anti-apoptosis in rats. Mol Biol Rep. (2014) 41:555–61. doi: 10.1007/s11033-013-2891-x. PMID: 24338162

[73] Du J-K Cong B-H Yu Q Wang H Wang L Wang C-N . Upregulation of microRNA-22 contributes to myocardial ischemia-reperfusion injury by interfering with the mitochondrial function. Free Radical Biol Med. (2016) 96:406–17. doi: 10.1016/j.freeradbiomed.2016.05.006. PMID: 27174562

[74] Ko JH Kim SN . MicroRNA in acupuncture studies: does small RNA shed light on the biological mechanism of acupuncture? Evidence-Based Complementary Altern Medicine: eCAM. (2019) 2019:3051472. doi: 10.1155/2019/3051472. PMID: 31118954 PMC6500616

[75] Chen B Li M-Y Guo Y Zhao X Lim H-MC . Mast cell-derived exosomes at the stimulated acupoints activating the neuro-immune regulation. Chin J Integr Med. (2017) 23:878–80. doi: 10.1007/s11655-016-2269-8. PMID: 27650095

[76] Liu Y-P Yang Y-D Mou F-F Zhu J Li H Zhao T-T . Exosome-mediated miR-21 was involved in the promotion of structural and functional recovery effect produced by electroacupuncture in sciatic nerve injury. Oxid Med Cell Longevity. (2022) 2022:7530102. doi: 10.1155/2022/7530102. PMID: 35132352 PMC8817850

[77] Klein JD Wang XH . Electrically stimulated acupuncture increases renal blood flow through exosome-carried miR-181. Am J Physiol Renal Physiol. (2018) 315:F1542–9. doi: 10.1152/ajprenal.00259.2018. PMID: 30132347 PMC6336992

[78] Huang Y Yu M Kuma A Klein JD Wang Y Hassounah F . Downregulation of let-7 by electrical acupuncture increases protein synthesis in mice. Front Physiol. (2021) 12:697139. doi: 10.3389/fphys.2021.697139. PMID: 34489723 PMC8417904

[79] Xu S-Y Zeng C-L Ni S-M Peng Y-J . The angiogenesis effects of electro-acupuncture treatment via exosomal miR-210 in cerebral ischemia-reperfusion rats. Curr Neurovascular Res. (2022) 19:61–72. doi: 10.2174/1567202619666220321115412. PMID: 35319370

[80] Zhang S Jin T Wang L Liu W Zhang Y Zheng Y . Electro-acupuncture promotes the differentiation of endogenous neural stem cells via exosomal microRNA 146b after ischemic stroke. Front Cell Neurosci. (2020) 14:223. doi: 10.3389/fncel.2020.00223. PMID: 32792909 PMC7385414

[81] Kuang H Zhang C Zhang W Cai H Yang L Yuan N . Electroacupuncture improves intestinal motility through exosomal miR-34c-5p targeting SCF/c-kit signaling pathway in slow transit constipation model rats. Evidence-Based Complementary Altern Medicine: eCAM. (2022) 2022:8043841. doi: 10.1155/2022/8043841. PMID: 36133788 PMC9484875

[82] Zou Y Bhat OM Yuan X Li G Huang D Guo Y . Release and actions of inflammatory exosomes in pulmonary emphysema: potential therapeutic target of acupuncture. J Inflammation Res. (2021) 14:3501–21. doi: 10.2147/jir.s312385. PMID: 34335040 PMC8318722

[83] Xia X Ding Y Zhou C Zhang H Yang X Shen C . Electroacupuncture preconditioning attenuates myocardial ischemia-reperfusion injury in rats partially through Nrf2-mediated reduction of oxidative stress and pyroptosis. Am J Chin Med. (2025) 53:337–52. doi: 10.1142/s0192415x25500132. PMID: 40107884

[84] Fu Y Li J Wu S Wang H . Electroacupuncture pretreatment promotes angiogenesis via hypoxia-inducible factor 1α and vascular endothelial growth factor in a rat model of chronic myocardial ischemia. Acupuncture Medicine: J Br Med Acupuncture Soc. (2021) 39:367–75. doi: 10.1177/0964528420938378. PMID: 32811184

[85] Zhou J Zhang B Zhou X Zhang F Shu Q Wu Y . Electroacupuncture pretreatment mediates sympathetic nerves to alleviate myocardial ischemia-reperfusion injury via CRH neurons in the paraventricular nucleus of the hypothalamus. Chin Med. (2024) 19:43. doi: 10.1186/s13020-024-00916-y. PMID: 38448912 PMC10916233

[86] Wang N Ma J Ma Y Lu L Ma C Qin P . Electroacupuncture pretreatment mitigates myocardial ischemia/reperfusion injury via XBP1/GRP78/akt pathway. Front Cardiovasc Med. (2021) 8:629547. doi: 10.3389/fcvm.2021.629547. PMID: 34195232 PMC8236521

[87] Li X Wang L Ying X Zheng Y Tan Q Yu X . Electroacupuncture pre-treatment alleviates sepsis-induced cardiac inflammation and dysfunction by inhibiting the calpain-2/STAT3 pathway. Front Physiol. (2022) 13:961909. doi: 10.3389/fphys.2022.961909. PMID: 36160853 PMC9489935

[88] Han Y-L Chen S Peng X . Electroacupuncture pretreatment at neiguan (PC6) attenuates autophagy in rats with myocardial ischemia reperfusion through the phosphatidylinositol 3-kinase-akt-mammalian target of rapamycin pathway. J Tradit Chin Med = Chung Tsa Chih Ying Wen Pan. (2021) 41:455–62. doi: 10.19852/j.cnki.jtcm.2021.03.014 34114404

[89] Han Y Chen S Wang H Peng X-M . Electroacupuncture pretreatment regulates apoptosis of myocardial ischemia-reperfusion injury in rats through RhoA/p38MAPK pathway mediated by miR-133a-5p. Evidence-Based Complementary Altern Medicine: eCAM. (2021) 2021:8827891. doi: 10.1155/2021/8827891. PMID: 33763149 PMC7964106

[90] Xiao Y Ding L . Mechanistic study of electroacupuncture preconditioning in alleviating myocardial ischemia-reperfusion injury in rats: involvement of mTOR/ROS signaling pathway to inhibit ferroptosis. Int J Neurosci. (2025) 135:287–95. doi: 10.1080/00207454.2023.2299315. PMID: 38197187

[91] Zhang T Yang W-X Wang Y-L Yuan J Qian Y Sun Q-M . Electroacupuncture preconditioning attenuates acute myocardial ischemia injury through inhibiting NLRP3 inflammasome activation in mice. Life Sci. (2020) 248:117451. doi: 10.1016/j.lfs.2020.117451. PMID: 32088213

[92] Bai H Xu S-L Shi J-J Ding Y-P Liu Q-Q Jiang C-H . Electroacupuncture preconditioning protects against myocardial ischemia-reperfusion injury by modulating dynamic inflammatory response. Heliyon. (2023) 9:e19396. doi: 10.1016/j.heliyon.2023.e19396. PMID: 37809701 PMC10558356

