# Supplementary figures and images for: Electroacupuncture alleviates myocardial ischemia-reperfusion injury by targeting and inhibiting NLRP3 inflammasome-mediated cardiomyocyte pyroptosis via serum exosomal miR-22-3p
Source: Front Immunol. 2026 Jun 1;17:1824799. doi: 10.3389/fimmu.2026.1824799 (PMC13266310; doi:10.3389/fimmu.2026.1824799)

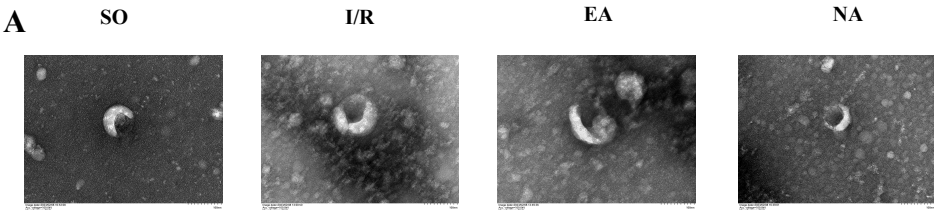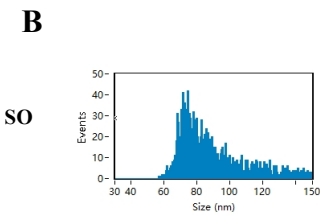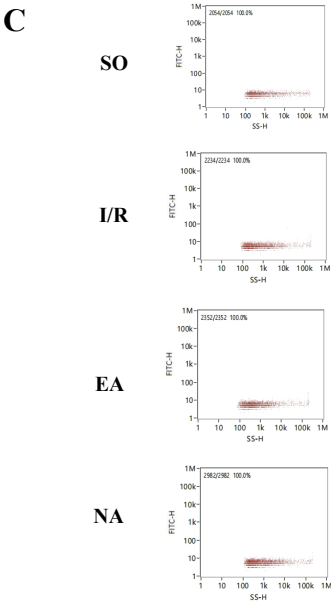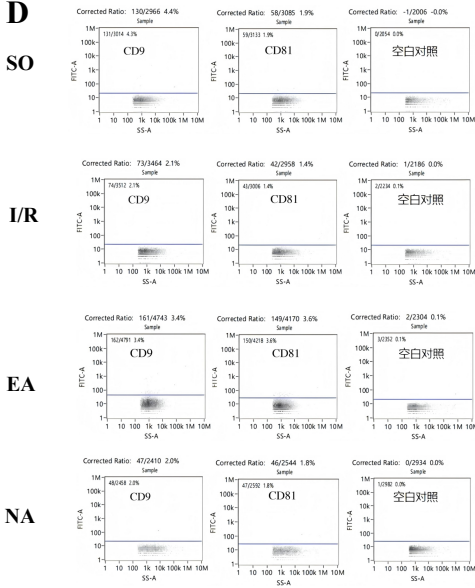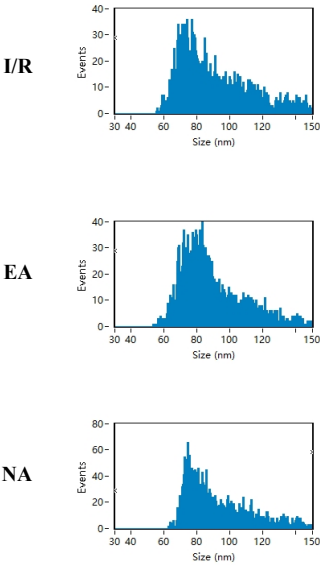

Supplement: Supplementary file 1 [file DataSheet1.pdf]

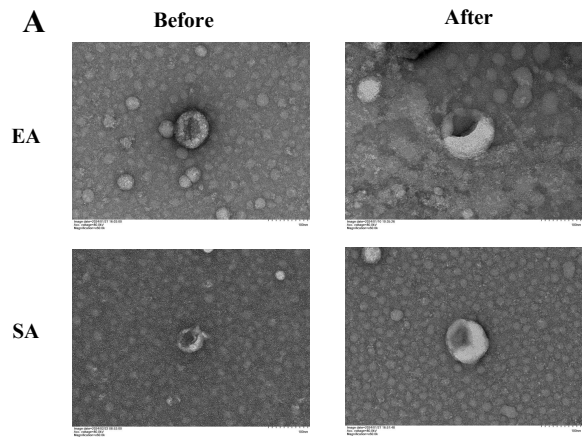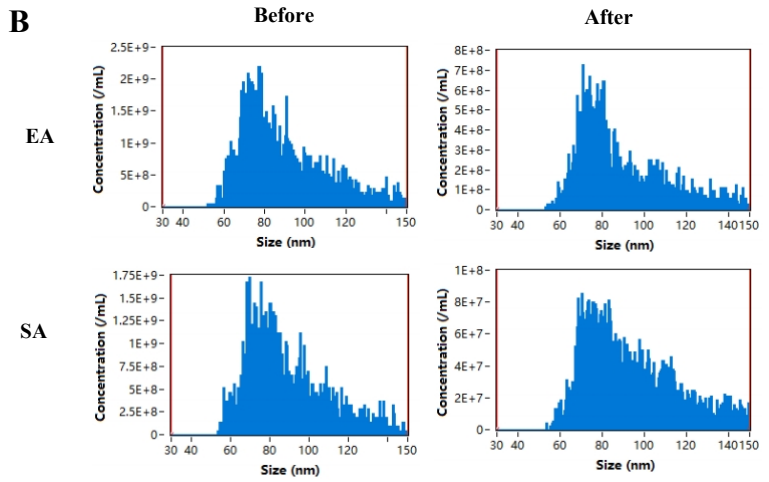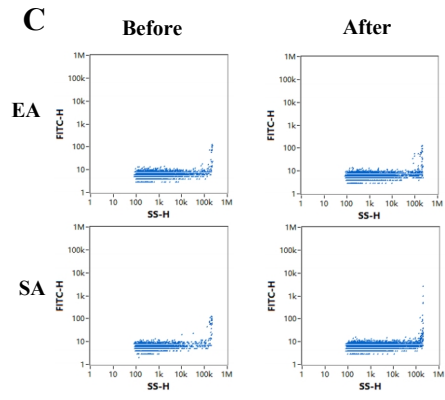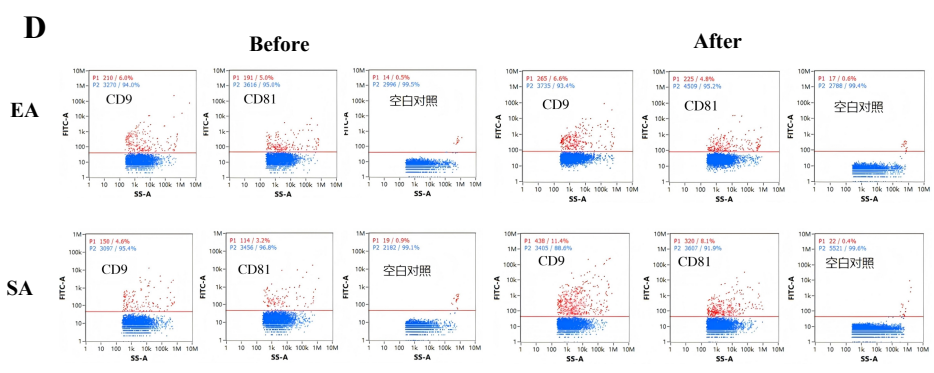

Supplement: Supplementary file 2 [file DataSheet2.pdf]
