# Supplementary material for: Electroacupuncture alleviates myocardial ischemia-reperfusion injury by targeting and inhibiting NLRP3 inflammasome-mediated cardiomyocyte pyroptosis via serum exosomal miR-22-3p
Source: Front Immunol. 2026 Jun 1;17:1824799. doi: 10.3389/fimmu.2026.1824799 (PMC13266310; doi:10.3389/fimmu.2026.1824799)
Supplement: Supplementary file 3 [file Table1.docx]

**Supplementary Table 1 Primer sequences**

| **Gene** | **Direction** | **Primer sequence (5'-3')** | **Product Length (bp)** |
| --- | --- | --- | --- |
| NLRP3 | FORWARD | GCCGTCTACGTCTTCTTCCTTTCC | 106 |
|  | REVERSE | CATCCGCAGCCAGTGAACAGAG |  |
| ASC | FORWARD | CAGTACCAGGCAGTTCGTGC | 134 |
|  | REVERSE | ACCAAGTAGGGCTGTGTTTGC |  |
| Caspase1 | FORWARD | ACACATTGAAGTGCCCAAGC | 74 |
|  | REVERSE | TTGTTTCTCTCCACGGCATG |  |
| GSDMD | FORWARD | GGCAACTTCCAAGTCTCCGATGTC | 149 |
|  | REVERSE | TGAGTCACACGCAGCATACACAC |  |
| IL-1β | FORWARD | AATCTCACAGCAGCATCTCGACAAG | 98 |
|  | REVERSE | TCCACGGGCAAGACATAGGTAGC |  |
| IL-18 | FORWARD | CGACCGAACAGCCAACGAATC | 80 |
|  | REVERSE | TCACAGCCAGTCCTCTTACTTCAC |  |
| β-Actin | FORWARD | TGTCACCAACTGGGACGATA | 165 |
|  | REVERSE | GGGGTGTTGAAGGTCTCAAA |  |
| miR-22-3p | FORWARD | ACTCCGGAAAGCTGCCAGTTG |  |
|  | REVERSE | ATCCAGTGCAGGGTCCGAGG |  |
|  | RT Primer | GTCGTATCCAGTGCAGGGTCCGAGGTATTCGCACTGGATACGACACAGTT |  |
| U6 | FORWARD | CTCGCTTCGGCAGCACA |  |
|  | REVERSE | AACGCTTCACGAATTTGCGT |  |

**Supplement Table 2 Main information table of miRNA differentially expressed in serum exosomes between model (I/R) group and sham operation (SO) group**

| **id** | **log2FoldChange** | **pval** | **Regulation** |
| --- | --- | --- | --- |
| miR-26a-5p | 1.249638461 | 5.6667E-05 | Up |
| let-7c-5p | 1.290137076 | 0.00015986 | Up |
| let-7a-5p | 1.147905792 | 0.000185714 | Up |
| miR-186-5p | 1.500107489 | 0.000194606 | Up |
| miR-1843b-5p | Inf | 0.000255735 | Up |
| let-7f-5p | 1.007271599 | 0.000708613 | Up |
| miR-652-3p | 1.297962738 | 0.000795385 | Up |
| miR-30c-5p | 1.202799574 | 0.00146865 | Up |
| miR-26b-5p | 1.069470482 | 0.001827353 | Up |
| miR-28-5p | 2.24722784 | 0.004887708 | Up |
| miR-144-5p | 1.338557911 | 0.015469324 | Up |
| miR-7a-5p | 1.178550583 | 0.019502317 | Up |
| let-7b-5p | 1.125239717 | 0.027278778 | Up |
| miR-1843a-5p | 2.732644926 | 0.03819367 | Up |
| miR-106b-3p | 1.520460816 | 0.038645259 | Up |
| miR-199a-3p | -1.933121229 | 6.62706E-07 | Down |
| miR-22-3p | -1.186306657 | 0.000261729 | Down |
| miR-10b-5p | -1.212050421 | 0.000280511 | Down |
| miR-487b-3p | -Inf | 0.000780234 | Down |
| miR-27b-3p | -1.058394398 | 0.002199132 | Down |
| miR-146a-5p | -1.035158614 | 0.00230818 | Down |
| miR-181a-5p | -1.103052621 | 0.003605711 | Down |
| miR-125b-1-3p | -Inf | 0.003649415 | Down |
| miR-99a-5p | -1.599821826 | 0.004584068 | Down |
| miR-125b-5p | -1.581100721 | 0.005747354 | Down |
| miR-214-3p | -1.900447881 | 0.007037613 | Down |
| miR-351-3p | -4.005514315 | 0.007808797 | Down |
| miR-193a-5p | -Inf | 0.008568668 | Down |
| miR-3594-3p | -Inf | 0.009598957 | Down |
| miR-29b-3p | -1.626762431 | 0.014340592 | Down |
| miR-653-3p | -4.419418377 | 0.017772977 | Down |
| miR-100-5p | -1.66823752 | 0.018493584 | Down |
| miR-3594-5p | -Inf | 0.025241533 | Down |
| miR-375-3p | -1.843761176 | 0.025896991 | Down |
| miR-192-5p | -1.185353204 | 0.028927469 | Down |
| miR-221-3p | -1.359414226 | 0.032366071 | Down |
| miR-34b-3p | -5.224920655 | 0.033542404 | Down |
| miR-149-5p | -1.429599045 | 0.036927244 | Down |
| miR-145-3p | -1.293109823 | 0.045651694 | Down |
| miR-9a-5p | -Inf | 0.046338315 | Down |
| miR-409a-3p | -Inf | 0.049115013 | Down |

**Supplement Table 3 Main information of miRNA differentially expressed in serum exosomes between EA group and model (I/R) group**

| **id** | **log2FoldChange** | **pval** | **Regulation** |
| --- | --- | --- | --- |
| miR-351-3p | 4.417919635 | 0.007261462 | Up |
| miR-196a-5p | Inf | 0.014145607 | Up |
| miR-184 | 2.490613946 | 0.023502082 | Up |
| miR-450a-5p | 2.046521727 | 0.023628626 | Up |
| miR-22-3p | 1.119400294 | 0.024544272 | Up |
| miR-125a-5p | 1.112026362 | 0.025264341 | Up |
| miR-196c-5p | Inf | 0.043212309 | Up |
| miR-1843b-5p | -8.451837205 | 0.007581972 | Down |
| miR-20a-5p | -1.700695011 | 0.015702252 | Down |
| miR-186-5p | -1.087681571 | 0.017149561 | Down |
| miR-6329 | -Inf | 0.022699088 | Down |

**Supplement Table 4 Main information of miRNA differentially expressed in serum exosomes between NA group and I/R group**

| **id** | **log2FoldChange** | **pval** | **Regulation** |
| --- | --- | --- | --- |
| miR-409a-3p | Inf | 0.000681718 | UP |
| miR-132-3p | Inf | 0.00734169 | UP |
| miR-429 | 4.564422412 | 0.023638678 | UP |
| miR-186-5p | -1.197234791 | 0.045846274 | down |

**Supplement Table 5 Main information of serum exosomes miRNA differentially expressed before and after treatment with AMI-PCI in EA group**

| **id** | **log2FoldChange** | **pval** | **Regulation** |
| --- | --- | --- | --- |
| miR-1-3p | 5.703490115 | 5.63011E-07 | Up |
| miR-9-5p | 4.408241386 | 5.2762E-06 | Up |
| miR-34c-5p | 5.810040817 | 6.31952E-06 | Up |
| miR-200b-3p | 4.995089308 | 1.57127E-05 | Up |
| miR-215-5p | 5.212152707 | 3.42425E-05 | Up |
| miR-200a-3p | 4.12661688 | 7.69875E-05 | Up |
| miR-301b-3p | 3.49957128 | 0.000563265 | Up |
| miR-217-5p | 7.833812269 | 0.00064805 | Up |
| miR-218-5p | 3.803722156 | 0.000796626 | Up |
| miR-22-3p | 2.541521812 | 0.000935306 | Up |
| miR-138-5p | 6.675938389 | 0.001000313 | Up |
| miR-129-5p | 3.563584241 | 0.001527957 | Up |
| miR-133a-3p | 3.191302446 | 0.001937128 | Up |
| miR-127-3p | 1.328155364 | 0.002835598 | Up |
| miR-133a-5p | 6.730256009 | 0.004132445 | Up |
| miR-196a-5p | 3.146553586 | 0.004442406 | Up |
| miR-143-3p | 1.654458463 | 0.005202081 | Up |
| miR-708-5p | 4.240530755 | 0.005356289 | Up |
| miR-206 | 3.987646811 | 0.005447175 | Up |
| miR-376b-3p | 2.957546596 | 0.007192948 | Up |
| miR-449a | 3.578352396 | 0.008172309 | Up |
| miR-377-3p | 3.196783073 | 0.008335652 | Up |
| miR-130a-5p | 4.18423359 | 0.013616492 | Up |
| miR-34b-5p | 5.432044223 | 0.013722944 | Up |
| miR-31-5p | 2.221964777 | 0.014018484 | Up |
| miR-200b-5p | 3.037097399 | 0.014935755 | Up |
| let-7c-5p | 1.180458421 | 0.015075597 | Up |
| miR-128-1-5p | 2.567875227 | 0.015660246 | Up |
| miR-124-3p | 6.102321407 | 0.016143506 | Up |
| miR-455-5p | 2.775741638 | 0.016334581 | Up |
| miR-6764-5p | 2.758169214 | 0.017176123 | Up |
| miR-137-3p | 6.214190551 | 0.01721994 | Up |
| miR-5584-5p | 4.467401187 | 0.018428696 | Up |
| miR-187-3p | 2.276097123 | 0.019346694 | Up |
| miR-216a-5p | 3.8252766 | 0.019702022 | Up |
| miR-34b-3p | 4.428270294 | 0.020208951 | Up |
| miR-30d-3p | 1.780707717 | 0.02030344 | Up |
| miR-1271-5p | 1.442717941 | 0.021184184 | Up |
| miR-5193 | 3.395137739 | 0.021645625 | Up |
| miR-493-5p | 1.330842017 | 0.022031391 | Up |
| miR-376c-3p | 1.620420457 | 0.022159032 | Up |
| miR-412-5p | 2.370752884 | 0.024478224 | Up |
| miR-6735-5p | 3.437882378 | 0.024543012 | Up |
| miR-212-5p | 5.51132833 | 0.027482162 | Up |
| miR-3648 | 4.977641965 | 0.028314225 | Up |
| miR-1469 | 2.537955084 | 0.029890352 | Up |
| miR-6825-5p | 3.926266227 | 0.029970527 | Up |
| miR-100-5p | 2.188940907 | 0.030633094 | Up |
| miR-125b-5p | 1.329768174 | 0.032271505 | Up |
| miR-411-5p | 1.263834076 | 0.034518196 | Up |
| miR-545-5p | 3.828344074 | 0.034548614 | Up |
| miR-330-3p | 1.285428756 | 0.035140608 | Up |
| miR-3174 | 4.085395076 | 0.038351776 | Up |
| miR-6842-5p | 3.256011491 | 0.039039348 | Up |
| miR-515-5p | 3.816060252 | 0.03945272 | Up |
| miR-378a-5p | 2.071752319 | 0.041335881 | Up |
| miR-1256 | 4.144761327 | 0.043106646 | Up |
| miR-379-5p | 1.00845288 | 0.044565611 | Up |
| miR-186-3p | 2.000607059 | 0.04712954 | Up |
| miR-342-5p | 1.448980579 | 0.047598346 | Up |
| miR-133b | 2.989596621 | 0.04898114 | Up |
| miR-552-5p | 3.3924589 | 0.049280629 | Up |
| miR-208b-3p | -6.641915178 | 4.27673E-06 | Down |
| miR-320d | -2.408739912 | 0.000122781 | Down |
| miR-1246 | -2.216444643 | 0.000203593 | Down |
| miR-320c | -1.748182025 | 0.000463577 | Down |
| miR-331-5p | -5.590252408 | 0.000570672 | Down |
| miR-320b | -1.690671793 | 0.000729745 | Down |
| miR-1290 | -1.980154476 | 0.000981206 | Down |
| let-7d-3p | -1.376213755 | 0.002155545 | Down |
| miR-6089 | -5.764779926 | 0.002549984 | Down |
| miR-424-5p | -1.820745533 | 0.002923412 | Down |
| miR-4732-5p | -1.721384698 | 0.003554368 | Down |
| miR-6767-5p | -4.040895371 | 0.004298811 | Down |
| miR-483-3p | -1.412911803 | 0.004648687 | Down |
| miR-193b-5p | -3.129177013 | 0.00514814 | Down |
| miR-483-5p | -1.604479753 | 0.006068112 | Down |
| miR-1268a | -2.857050093 | 0.006757531 | Down |
| miR-10400-5p | -2.559351099 | 0.007003599 | Down |
| miR-1306-5p | -1.404444318 | 0.007158473 | Down |
| miR-378g | -3.825868472 | 0.007558771 | Down |
| miR-10b-3p | -1.682403405 | 0.008845612 | Down |
| miR-1268b | -2.698560103 | 0.009881706 | Down |
| miR-548ay-3p | -4.617627395 | 0.01035444 | Down |
| miR-1291 | -5.138533306 | 0.013512325 | Down |
| miR-548u | -4.956824367 | 0.013776158 | Down |
| miR-3173-5p | -2.107103073 | 0.017663594 | Down |
| miR-4488 | -3.421770971 | 0.018746181 | Down |
| miR-3150b-3p | -3.329786125 | 0.019446793 | Down |
| miR-1288-3p | -4.443342551 | 0.019718054 | Down |
| miR-551a | -3.61282841 | 0.021694134 | Down |
| miR-4777-3p | -4.179428045 | 0.022044857 | Down |
| miR-9903 | -3.545959271 | 0.022744263 | Down |
| miR-3163 | -4.512253457 | 0.028134594 | Down |
| miR-4440 | -5.006665812 | 0.029330793 | Down |
| miR-582-3p | -2.605181 | 0.030211659 | Down |
| miR-2276-3p | -3.052980114 | 0.030484482 | Down |
| miR-6781-5p | -3.965966648 | 0.03131227 | Down |
| miR-4492 | -3.280853538 | 0.032934598 | Down |
| miR-4786-5p | -3.57455179 | 0.033636538 | Down |
| miR-204-3p | -3.966475944 | 0.033741191 | Down |
| miR-122b-5p | -3.789427104 | 0.035582678 | Down |
| miR-378a-3p | -1.159143363 | 0.036481552 | Down |
| miR-208a-3p | -4.045224997 | 0.039808588 | Down |
| miR-499a-5p | -2.220242945 | 0.042305585 | Down |
| miR-450a-2-3p | -3.834008024 | 0.042351659 | Down |
| miR-3940-3p | -2.920651448 | 0.042789441 | Down |
| miR-138-1-3p | -3.969655473 | 0.04401279 | Down |
| miR-335-5p | -1.015032507 | 0.045662629 | Down |
| miR-664a-5p | -1.101456592 | 0.048716528 | Down |
| miR-12136 | -1.371872916 | 0.049013605 | Down |
| miR-5189-3p | -2.950474712 | 0.04931957 | Down |

**Supplement Table 6 Main information of serum exosomes miRNA differentially expressed before and after treatment with AMI-PCI in sham electroacupuncture (SA) group**

| **id** | **log2FoldChange** | **pval** | **Regulation** |
| --- | --- | --- | --- |
| miR-3065-3p | 2.57325064 | 0.001374972 | Up |
| miR-142-5p | 1.212082568 | 0.001508063 | Up |
| miR-548z | 2.513471854 | 0.003598159 | Up |
| miR-548h-3p | 2.513513341 | 0.003602985 | Up |
| miR-4700-5p | 5.935147657 | 0.008565434 | Up |
| miR-150-5p | 1.131886297 | 0.008933065 | Up |
| miR-503-5p | 1.204818358 | 0.009379671 | Up |
| miR-642a-3p | 3.877845308 | 0.012821165 | Up |
| miR-30e-3p | 1.173709653 | 0.012845804 | Up |
| miR-210-3p | 1.60646252 | 0.013347539 | Up |
| miR-1277-3p | 3.579331593 | 0.015496489 | Up |
| miR-296-3p | 2.365225626 | 0.015751414 | Up |
| miR-3605-3p | 1.762167612 | 0.016331572 | Up |
| miR-1226-3p | 1.7081386 | 0.016592002 | Up |
| miR-873-5p | 1.802206249 | 0.017019691 | Up |
| miR-1250-5p | 1.981900913 | 0.0209601 | Up |
| miR-95-3p | 1.23303153 | 0.021539751 | Up |
| miR-4732-3p | 1.281715069 | 0.022117453 | Up |
| miR-361-3p | 1.003628689 | 0.022877733 | Up |
| miR-6859-5p | 2.829153094 | 0.027780681 | Up |
| miR-130b-3p | 1.174637613 | 0.029082691 | Up |
| miR-3202 | 3.553064179 | 0.03180846 | Up |
| miR-6802-5p | 4.121917621 | 0.042351943 | Up |
| miR-1273c | 3.619426666 | 0.04490934 | Up |
| miR-6503-3p | 3.344501646 | 0.045495032 | Up |
| miR-6842-5p | 1.649634155 | 0.048205068 | Up |
| miR-20b-5p | 1.028658578 | 0.049054404 | Up |
| miR-133a-3p | -3.178037728 | 5.31E-08 | Down |
| miR-206 | -3.709313025 | 3.98E-05 | Down |
| miR-490-3p | -5.08099302 | 0.00012549 | Down |
| miR-208a-3p | -6.296048594 | 0.000150717 | Down |
| miR-378i | -4.639630456 | 0.000260371 | Down |
| miR-34c-5p | -5.057676508 | 0.00043499 | Down |
| miR-208b-3p | -3.254163836 | 0.000681642 | Down |
| miR-548a-3p | -3.595736957 | 0.001167721 | Down |
| miR-423-5p | -1.320470868 | 0.001352039 | Down |
| miR-499a-5p | -3.180164917 | 0.001770507 | Down |
| miR-9-5p | -1.572209526 | 0.004545553 | Down |
| miR-7704 | -5.598613117 | 0.005590464 | Down |
| miR-133a-5p | -4.399259159 | 0.006881752 | Down |
| miR-4446-3p | -3.102650556 | 0.007613432 | Down |
| miR-4738-3p | -2.478141719 | 0.007730466 | Down |
| miR-22-3p | -1.119781484 | 0.00935243 | Down |
| miR-1298-5p | -4.303443155 | 0.012483132 | Down |
| miR-4535 | -4.808410148 | 0.014522662 | Down |
| miR-1469 | -2.254538773 | 0.022171232 | Down |
| miR-205-5p | -2.125090669 | 0.022902107 | Down |
| miR-200b-5p | -2.54178107 | 0.024552931 | Down |
| miR-99b-3p | -1.953233956 | 0.025669917 | Down |
| miR-483-3p | -2.329718868 | 0.026566739 | Down |
| miR-665 | -3.009377808 | 0.026699671 | Down |
| miR-363-3p | -1.347367581 | 0.027230987 | Down |
| miR-145-3p | -2.407643026 | 0.028646117 | Down |
| miR-3138 | -1.514621412 | 0.032267142 | Down |
| miR-133b | -2.391021623 | 0.034348883 | Down |
| miR-1-3p | -1.030972952 | 0.035044378 | Down |
| miR-136-5p | -2.315572378 | 0.040195644 | Down |
| miR-200a-3p | -1.444766853 | 0.042731631 | Down |
| miR-96-5p | -1.494550217 | 0.043235384 | Down |
| miR-6810-5p | -3.517396469 | 0.04358064 | Down |
| miR-421 | -1.212217025 | 0.044219783 | Down |
| miR-365b-5p | -3.689099412 | 0.044922291 | Down |
| miR-9-3p | -1.503499667 | 0.046515055 | Down |
| miR-6741-3p | -1.691817571 | 0.04833246 | Down |

**Supplement Table 7 Molecular Screening Table for Key Serum Exosomes Upregulated by Electroacupuncture (EA)**

| EA_Up | SA_Up | EA_Up\|SA_Up |
| --- | --- | --- |
| miR-1-3p | miR-3065-3p | miR-6842-5p |
| miR-9-5p | miR-142-5p |  |
| miR-34c-5p | miR-548z |  |
| miR-200b-3p | miR-548h-3p |  |
| miR-215-5p | miR-4700-5p |  |
| miR-200a-3p | miR-150-5p |  |
| miR-301b-3p | miR-503-5p |  |
| miR-217-5p | miR-642a-3p |  |
| miR-218-5p | miR-30e-3p |  |
| miR-22-3p | miR-210-3p |  |
| miR-138-5p | miR-1277-3p |  |
| miR-129-5p | miR-296-3p |  |
| miR-133a-3p | miR-3605-3p |  |
| miR-127-3p | miR-1226-3p |  |
| miR-133a-5p | miR-873-5p |  |
| miR-196a-5p | miR-1250-5p |  |
| miR-143-3p | miR-95-3p |  |
| miR-708-5p | miR-4732-3p |  |
| miR-206 | miR-361-3p |  |
| miR-376b-3p | miR-6859-5p |  |
| miR-449a | miR-130b-3p |  |
| miR-377-3p | miR-3202 |  |
| miR-130a-5p | miR-6802-5p |  |
| miR-34b-5p | miR-1273c |  |
| miR-31-5p | miR-6503-3p |  |
| miR-200b-5p | miR-20b-5p |  |
| let-7c-5p |  |  |
| miR-128-1-5p |  |  |
| miR-124-3p |  |  |
| miR-455-5p |  |  |
| miR-6764-5p |  |  |
| miR-137-3p |  |  |
| miR-5584-5p |  |  |
| miR-187-3p |  |  |
| miR-216a-5p |  |  |
| miR-34b-3p |  |  |
| miR-30d-3p |  |  |
| miR-1271-5p |  |  |
| miR-5193 |  |  |
| miR-493-5p |  |  |
| miR-376c-3p |  |  |
| miR-412-5p |  |  |
| miR-6735-5p |  |  |
| miR-212-5p |  |  |
| miR-3648 |  |  |
| miR-1469 |  |  |
| miR-6825-5p |  |  |
| miR-100-5p |  |  |
| miR-125b-5p |  |  |
| miR-411-5p |  |  |
| miR-545-5p |  |  |
| miR-330-3p |  |  |
| miR-3174 |  |  |
| miR-515-5p |  |  |
| miR-378a-5p |  |  |
| miR-1256 |  |  |
| miR-379-5p |  |  |
| miR-186-3p |  |  |
| miR-342-5p |  |  |
| miR-133b |  |  |
| miR-552-5p |  |  |

**Supplement Table 8 Molecular Screening Table of Key Serum Exosomes MiRNA Downregulated by Electroacupuncture (EA)**

| EA_Down | SA_Down | EA_Down\|SA_Down |
| --- | --- | --- |
| miR-320d | miR-133a-3p | miR-208b-3p |
| miR-1246 | miR-206 | miR-483-3p |
| miR-320c | miR-490-3p | miR-208a-3p |
| miR-331-5p | miR-378i | miR-499a-5p |
| miR-320b | miR-34c-5p |  |
| miR-1290 | miR-548a-3p |  |
| let-7d-3p | miR-423-5p |  |
| miR-6089 | miR-9-5p |  |
| miR-424-5p | miR-7704 |  |
| miR-4732-5p | miR-133a-5p |  |
| miR-6767-5p | miR-4446-3p |  |
| miR-193b-5p | miR-4738-3p |  |
| miR-483-5p | miR-22-3p |  |
| miR-1268a | miR-1298-5p |  |
| miR-10400-5p | miR-4535 |  |
| miR-1306-5p | miR-1469 |  |
| miR-378g | miR-205-5p |  |
| miR-10b-3p | miR-200b-5p |  |
| miR-1268b | miR-99b-3p |  |
| miR-548ay-3p | miR-665 |  |
| miR-1291 | miR-363-3p |  |
| miR-548u | miR-145-3p |  |
| miR-3173-5p | miR-3138 |  |
| miR-4488 | miR-133b |  |
| miR-3150b-3p | miR-1-3p |  |
| miR-1288-3p | miR-136-5p |  |
| miR-551a | miR-200a-3p |  |
| miR-4777-3p | miR-96-5p |  |
| miR-9903 | miR-6810-5p |  |
| miR-3163 | miR-421 |  |
| miR-4440 | miR-365b-5p |  |
| miR-582-3p | miR-9-3p |  |
| miR-2276-3p | miR-6741-3p |  |
| miR-6781-5p |  |  |
| miR-4492 |  |  |
| miR-4786-5p |  |  |
| miR-204-3p |  |  |
| miR-122b-5p |  |  |
| miR-378a-3p |  |  |
| miR-450a-2-3p |  |  |
| miR-3940-3p |  |  |
| miR-138-1-3p |  |  |
| miR-335-5p |  |  |
| miR-664a-5p |  |  |
| miR-12136 |  |  |
| miR-5189-3p |  |  |
